# CPT1A as a metabolic gatekeeper linking fatty acid oxidation, ferroptosis resistance, and breast cancer stemness

**DOI:** 10.3389/fonc.2026.1959579

**Published:** 2026-09-24

**Authors:** Feng Zeng, Xinghan Chen, Kaiguo Long, Benjun Yu, Yuhuai Cai, Yulan Cai, Chuyu Liu

**Affiliations:** 1 Thyroid and Breast Surgery, the Second Affiliated Hospital of Zunyi Medical University, Zunyi, China; 2 Department of Endocrinology and Metabolism, Affiliated Hospital of Zunyi Medical University, Zunyi, China; 3 Department of Endocrinology, the Second Affiliated Hospital of Zunyi Medical University, Zunyi, China

**Keywords:** breast cancer, breast cancer stem cells, CPT1a, fatty acid oxidation, ferroptosis, lipid peroxidation, metabolic plasticity, therapy resistance

## Abstract

Breast cancer stem-like cells sustain residual disease, metastatic recurrence and resistance to systemic and local therapies. Their defining metabolic feature is not a fixed preference for glycolysis or oxidative phosphorylation, but the capacity to reallocate substrates while preserving energy and redox homeostasis. Carnitine palmitoyltransferase 1A (CPT1A), which controls mitochondrial entry of long-chain fatty acids, is increasingly linked to breast cancer stemness, metastatic competence, radioresistance and endocrine resistance. Ferroptosis creates a complementary therapeutic opportunity because stem-like cells depend on tightly regulated iron, phospholipid and antioxidant metabolism. Yet the direct relationship between CPT1A and ferroptosis in breast cancer stem cells remains incompletely resolved. Here, we distinguish direct causal evidence in breast cancer from pathway-level observations and cross-cancer mechanisms, and propose that CPT1A functions as a context-dependent metabolic gatekeeper. By allocating long-chain fatty acids among mitochondrial oxidation, neutral-lipid storage and peroxidation-prone membrane phospholipids, CPT1A may coordinate adenosine triphosphate (ATP) supply, redox buffering, phenotypic plasticity and the ferroptosis threshold. Direct breast cancer evidence links CPT1A to stemness in miR-328-3p-regulated, luminal and radiation-selected models, and to ferroptosis resistance downstream of peroxisome proliferator-activated receptor-γ (PPARγ) in triple-negative breast cancer (TNBC), whereas CPT1B contributes to leptin–STAT3-driven stemness and is co-regulated with CPT1A following OVOL2 loss. Breast cancer stem-cell ferroptosis is independently constrained by DKK1–SLC7A11, ZMYND8-dependent activation of nuclear factor erythroid 2-related factor 2 (NRF2), lysosomal iron handling and hypoxia-adapted cysteine metabolism. We integrate these findings into a subtype- and treatment-aware framework for combining CPT1A inhibition with ferroptosis induction. Critical barriers include CPT1 isoform specificity, pharmacological off-target effects, tumor–host metabolic interactions, normal-tissue toxicity and the absence of validated functional biomarkers. Resolving these issues will determine whether the CPT1A–ferroptosis–stemness axis can be translated from a mechanistic model into a therapeutic vulnerability.

## 1 Introduction

### 1.1 Breast cancer recurrence remains a problem of residual cell survival

Despite substantial advances in local and systemic treatment, recurrence remains the principal cause of breast cancer-related death. Its timing differs across molecular subtypes: triple-negative and human epidermal growth factor receptor 2 (HER2)-positive tumors tend to relapse earlier, whereas estrogen receptor (ER)-positive disease may recur after a prolonged clinically silent interval. In women with estrogen receptor-positive early breast cancer who remained disease-free after 5 years of endocrine therapy, distant recurrences continued to accumulate over the subsequent 15 years (1). This long tail of relapse is difficult to reconcile with the idea that apparent clinical remission necessarily reflects complete tumor eradication.

A more plausible explanation is that a small population of malignant cells survives initial treatment and persists below the threshold of conventional detection. These cells may remain at the primary site, circulate intermittently, or lodge within distant tissue niches, where they can enter dormancy or sustain limited proliferation for extended periods. The observation that some patients relapse despite achieving a pathological complete response after neoadjuvant therapy supports this view (2). Likewise, circulating tumor DNA can reveal molecular residual disease well before radiological recurrence becomes evident (3), indicating that occult disease may persist long after treatment has ended.

Residual cells should not be regarded as passive remnants of the original tumor. They are the product of strong therapeutic selection and are often enriched for traits that favor survival under adverse conditions, including reversible quiescence, efficient stress adaptation, redox control, and flexible nutrient use. Dormant disseminated tumor cells may also rely on specific survival pathways that are absent or less important in actively proliferating tumor cells (4). These features overlap substantially with the functional properties attributed to breast cancer stem cells, particularly tumor re-initiation, metastatic competence, and resistance to treatment. Recurrence may therefore be better understood as a problem of residual cell state and survival capacity, rather than residual tumor burden alone.

### 1.2 Breast cancer stem cells are metabolic states rather than a fixed population

Breast cancer stem cells (BCSCs) were initially described as a discrete subpopulation with self-renewal and tumor-initiating capacity. This hierarchical model remains useful, but it cannot fully explain the marked plasticity observed in breast tumors. CD44^high/CD24^low and aldehyde dehydrogenase (ALDH)^high cells represent partially distinct stem-like states, and transitions between them can occur in response to hypoxia, inflammation, treatment, and changes in the surrounding niche (5). Stemness is therefore unlikely to be a permanent attribute restricted to a fixed cell fraction. It is better viewed as a reversible state that can be acquired or lost as tumor cells adapt to environmental pressure.

Metabolism is closely involved in this process. Some BCSC populations preferentially use glycolysis, whereas others depend more heavily on mitochondrial respiration or fatty acid oxidation (6, 7). These differences probably reflect variation in molecular subtype, nutrient supply, treatment history, and metastatic location rather than inconsistency between studies. The common feature is not reliance on one pathway, but the ability to switch between metabolic programs while preserving redox balance and survival. This metabolic flexibility may help explain why depletion of one phenotypically defined BCSC population does not always prevent the later re-emergence of stem-like cells.

The operational definition of a BCSC is central to this interpretation. The original CD44high/CD24low and ALDH-based studies identified populations enriched for tumor initiation, but neither marker establishes an immutable lineage (62, 63). Subsequent work showed that breast cancer populations can return to a phenotypic equilibrium through stochastic state transitions, that non-stem cells can reacquire stem-like properties, and that chromatin at the ZEB1 locus can poise cells for such conversion (64–66). Epithelial–mesenchymal transition (EMT) can generate stem-associated traits, yet breast tumors traverse several intermediate epithelial–mesenchymal states rather than a single binary switch (67–70). Single-cell analysis of early metastatic breast cancer further identified a stem-like program that diminished as metastatic burden increased (71). The useful unit of analysis is therefore a state with demonstrable self-renewal and tumor-initiation capacity, not a marker-positive fraction considered in isolation.

### 1.3 Ferroptosis represents both a vulnerability and an adaptive challenge

Ferroptosis is an iron-dependent form of cell death driven by the accumulation of oxidized phospholipids. BCSCs may be particularly vulnerable to this process because their survival requires active iron handling, lipid metabolism, and antioxidant control. The same metabolic programs that support tumor initiation and persistence may become liabilities when lipid peroxide production exceeds the capacity of cellular defense systems.

This vulnerability is not fixed. Stem-like breast cancer cells can strengthen ferroptosis resistance by increasing cystine uptake, glutathione synthesis, lipid peroxide detoxification, and iron sequestration. DKK1 has been shown to protect metastatic breast cancer cells through SLC7A11-dependent suppression of lipid peroxidation (8), whereas ZMYND8 supports ALDH^high BCSCs by reinforcing antioxidant defense and limiting intracellular iron accumulation (9). These findings suggest that ferroptosis resistance can become part of the stem-like state itself. The relevant question is therefore not whether BCSCs are inherently sensitive or resistant to ferroptosis, but how their metabolic state determines the threshold at which lipid oxidative stress becomes lethal.

### 1.4 Why CPT1A may represent the missing metabolic link

Carnitine palmitoyltransferase 1A (CPT1A) regulates the entry of long-chain fatty acids into mitochondria and is a major determinant of fatty acid oxidation. In breast cancer, CPT1A has been linked to several features of residual and aggressive disease. The miR-328-3p–CPT1A axis regulates fatty acid oxidation, stem-like properties, spheroid formation, and metastatic capacity (10). CPT1A also supports colony and mammosphere formation in hormone receptor-positive breast cancer (11), while CPT1A/CPT2-dependent fatty acid oxidation is enhanced in radioresistant breast cancer cells and radiation-derived stem-like populations (12).

These studies place CPT1A at the intersection of mitochondrial adaptation, stemness, and treatment persistence. Direct evidence connecting CPT1A with ferroptosis in breast cancer is more limited, but has begun to emerge. In triple-negative breast cancer (TNBC) models, disruption of peroxisome proliferator-activated receptor-α (PPARα)–CPT1A-dependent fatty acid metabolism increased lipid peroxidation and ferroptotic cell death, whereas CPT1A restoration partially reversed these effects (13). This finding supports a role for CPT1A in ferroptosis control, although the mechanism has not yet been demonstrated specifically in functionally defined BCSCs. We therefore propose that CPT1A may act as a metabolic gatekeeper by influencing how fatty acids are distributed between mitochondrial oxidation, neutral-lipid storage, and membrane phospholipid synthesis. Through this position, CPT1A could simultaneously affect energy production, redox buffering, the availability of oxidizable lipid substrates, and the maintenance of stem-like states.

### 1.5 Scope and objectives of this review

This review examines CPT1A as a potential metabolic link between fatty acid oxidation, ferroptosis resistance, and breast cancer stemness. We first discuss how CPT1A-dependent metabolism supports stem-like and therapy-persistent cell states, and then summarize the mechanisms through which BCSCs limit iron-dependent lipid damage. These findings are integrated into a fatty acid-partitioning model in which CPT1A may influence whether fatty acids are directed toward mitochondrial oxidation, lipid storage, or ferroptosis-sensitive membrane phospholipids.

The review also considers differences across breast cancer subtypes, treatment contexts, and metastatic sites, and evaluates the therapeutic implications of combining CPT1A inhibition with ferroptosis-inducing strategies. Because a complete CPT1A–ferroptosis–stemness pathway has not yet been established in a single breast cancer model, direct breast cancer evidence, mechanistic findings from other cancers, and hypothesis-generating interpretations are distinguished throughout.

## 2 Evidence hierarchy and interpretive framework

The literature linking CPT1A, fatty acid oxidation, ferroptosis, and breast cancer stemness is uneven. Several components of this axis have been studied directly in breast cancer, whereas others are supported mainly by observations in different tumor types or by metabolic inference. Treating these findings as equivalent would create an apparently complete pathway that is not yet fully demonstrated. We therefore use a four-level framework to indicate how strongly each mechanistic conclusion is supported.

Level I evidence refers to direct causal studies in breast cancer models. These studies manipulate CPT1A genetically or with adequately controlled pharmacological approaches and assess relevant functional consequences, such as fatty acid oxidation, lipid peroxidation, ferroptotic death, mammosphere formation, tumor initiation, or treatment resistance. Rescue experiments and orthogonal genetic and metabolic validation further strengthen this level of evidence.

Level II evidence includes breast cancer studies that establish biological relevance but do not complete the causal chain. Examples include associations between CPT1A expression and clinical outcome, enrichment of CPT1A in resistant or stem-like populations, changes in mammosphere formation, and treatment-response phenotypes without direct measurement of fatty acid oxidation or rigorous confirmation of ferroptosis. Such studies identify plausible relationships, but they cannot determine whether CPT1A is the primary driver or one component of a broader adaptive program.

Level III evidence comprises mechanistic findings established in other malignancies. For example, the CPT1A–c-Myc–nuclear factor erythroid 2-related factor 2 (NRF2)/glutathione peroxidase 4 (GPX4) network described in non-breast cancer stem-like cells (44) provides a useful model for understanding how fatty acid oxidation might be coupled to ferroptosis resistance. These findings can guide hypothesis development, but they should not be presented as established mechanisms in breast cancer without direct validation.

Level IV evidence refers to mechanisms inferred from metabolic principles, multi-omics associations, pathway enrichment, or indirect relationships among fatty acid utilization, antioxidant defense, and membrane lipid remodeling. These interpretations are valuable when they generate experimentally testable questions, but they remain provisional.

This framework is not intended as a formal clinical evidence-grading system. The level is assigned to a specific mechanistic relationship rather than to an entire study. A single report may therefore provide Level I evidence for one link, such as CPT1A-dependent fatty acid oxidation, but only Level II or Level IV support for its proposed connection with breast cancer stemness or ferroptosis. This distinction is maintained throughout the review to prevent cross-cancer findings and metabolic plausibility from being mistaken for direct breast cancer evidence (**Table 1**).

**Table 1:** Evidence hierarchy for the CPT1A–ferroptosis–stemness axis.

Evidence level	Definition	Minimum supporting evidence	Permitted interpretation	Principal limitation
Level I	Direct causal evidence in breast cancer	Genetic CPT1A manipulation with functional FAO, ferroptosis, stemness or treatment-response assays; preferably rescue	Supports causality within the tested breast cancer model	May remain subtype-, model- or treatment-specific
Level II	Associative or partially mechanistic breast cancer evidence	Clinical association, resistant models, mammosphere or marker changes, or incomplete pathway validation	Supports biological relevance and a plausible link	Does not establish a complete causal pathway
Level III	Direct mechanistic evidence from other malignancies	Genetic or pharmacological intervention with mechanistic rescue outside breast cancer	Supports cross-cancer plausibility and hypothesis development	Requires direct validation in breast cancer
Level IV	Inferential or hypothesis-generating evidence	Omics correlations, pathway enrichment, indirect interactions or metabolic reasoning	Supports a testable conceptual model	Cannot establish causality

## 3 CPT1A-centered fatty acid metabolism in breast cancer

### 3.1 The carnitine shuttle and mitochondrial fatty acid oxidation

Long-chain fatty acids entering a breast cancer cell through uptake, *de novo* synthesis or lipid-droplet lipolysis are first activated to fatty acyl-CoAs. This pool sits at a genuine metabolic branch point: it can be oxidized in mitochondria, esterified into neutral lipids, or incorporated into membrane phospholipids. The relative use of these routes, rather than total lipid abundance alone, determines whether fatty acids primarily support energy production, provide structural lipids, or supply substrates for peroxidation.

Because long-chain fatty acyl-CoAs do not cross the inner mitochondrial membrane directly, fatty acid oxidation (FAO) requires the carnitine shuttle. CPT1A, situated in the outer mitochondrial membrane, transfers the acyl group to carnitine; carnitine-acylcarnitine translocase (CACT/SLC25A20) then transports acylcarnitine across the inner membrane, and carnitine palmitoyltransferase 2 (CPT2) regenerates fatty acyl-CoA in the matrix (14, 15). Subsequent β-oxidation yields acetyl-CoA, NADH and FADH_2_ for the tricarboxylic-acid cycle and oxidative phosphorylation (OXPHOS). CPT1A therefore controls mitochondrial fatty-acid entry; it does not itself generate adenosine triphosphate (ATP). In hormone receptor-positive breast cancer, CPT1A perturbation reduces fatty-acid oxidation and compromises tumor-cell growth, survival and mammosphere formation (11).

CPT1A should be distinguished from CPT1B, a closely related outer-mitochondrial-membrane isoform that catalyzes the same acylcarnitine-forming reaction but differs in physiological distribution and regulation. CPT1A is broadly expressed and predominates in liver and several other metabolically diverse tissues, whereas CPT1B is enriched in tissues with high and fluctuating fatty-acid oxidative demand, particularly skeletal muscle, heart and brown adipose tissue (14). CPT1B has a lower affinity for carnitine and is substantially more sensitive to inhibition by malonyl-CoA than CPT1A, suggesting that the two isoforms operate within different substrate and regulatory ranges despite their shared biochemical function (14). This distinction is also relevant to breast cancer. Direct studies implicate CPT1A in miR-328-3p-regulated metastatic stemness, hormone receptor-positive tumor fitness and radiation-selected fatty-acid oxidation (10–12). By contrast, adipocyte-derived leptin activates a JAK/STAT3–CPT1B pathway that promotes breast cancer stem-cell self-renewal and chemoresistance (7), while loss of OVOL2 can induce both CPT1A and CPT1B through STAT3 in triple-negative breast cancer (28). CPT1B-dependent findings therefore support the broader importance of mitochondrial fatty-acid oxidation in breast cancer but should not automatically be interpreted as evidence for a CPT1A-specific mechanism.

Fatty acids excluded from mitochondria are not automatically destined for peroxidation. They may be buffered in triacylglycerol-rich lipid droplets or remodeled into membrane phospholipids. acyl-CoA synthetase long-chain family member 4 (ACSL4)- and lysophosphatidylcholine acyltransferase 3 (LPCAT3)-dependent incorporation of polyunsaturated fatty acids (PUFAs) expands the phospholipid pool that can be oxidized during ferroptosis (35, 36), whereas monounsaturated fatty acid (MUFA) incorporation can be protective (41). Lipid droplets add a further temporal layer by sequestering fatty acids and releasing them during lipolysis. Long-chain fatty acids can therefore be directed toward mitochondrial oxidation, stored as triacylglycerol in lipid droplets, or incorporated into membrane phospholipids, with distinct consequences for energy production and lipid-peroxidation susceptibility (**Figure 1**).

**Figure 1:**
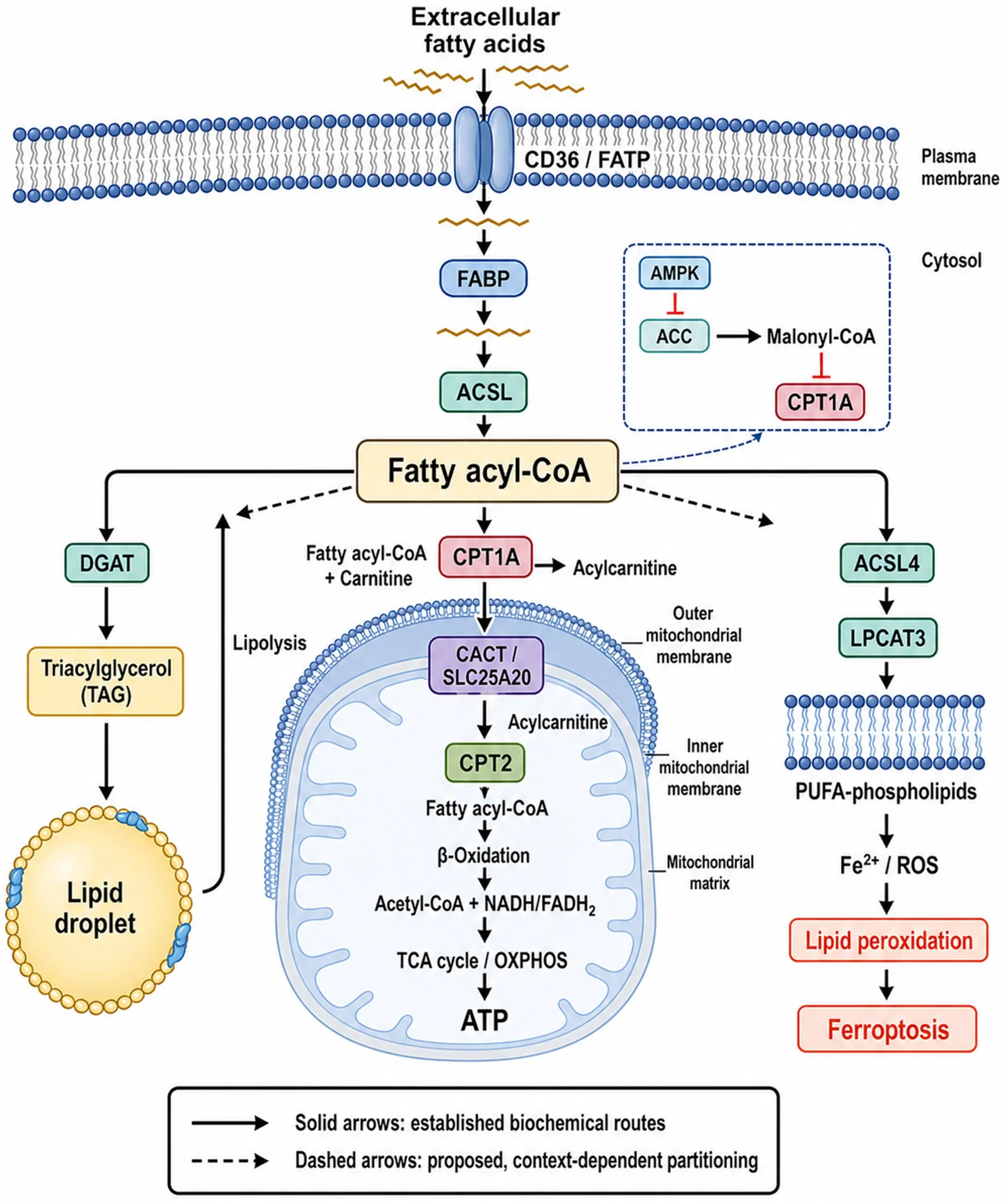
CPT1A-centered fatty acid trafficking and metabolic partitioning in breast cancer cells. Activated long-chain fatty acids may undergo CPT1A–CACT–CPT2-mediated mitochondrial transport and β-oxidation, DGAT-dependent storage as triacylglycerol in lipid droplets, or ACSL4/LPCAT3-dependent incorporation into PUFA-containing phospholipids. The latter pool supplies substrates for iron-dependent lipid peroxidation. Solid arrows indicate established biochemical routes; dashed arrows indicate proposed, context-dependent partitioning.

### 3.2 Regulation of CPT1A expression and activity

CPT1A is controlled on several timescales. Catalytic activity is acutely restrained by malonyl-CoA, linking mitochondrial fatty-acid entry to the balance between lipid synthesis and oxidation. During energy stress, AMP-activated protein kinase (AMPK)-mediated inhibition of acetyl-CoA carboxylase (ACC) lowers malonyl-CoA and relieves CPT1 inhibition; conversely, abundant malonyl-CoA can restrict flux despite unchanged CPT1A protein (14). Expression is therefore an incomplete surrogate for activity.

Longer-term regulation is imposed by genomic and transcriptional context. CPT1A lies within the chromosome 11q13–14 amplicon and is overexpressed in a subset of high-risk hormone receptor-positive tumors, in which genetic studies support a functional contribution to proliferation, colony formation and mammosphere growth (11). Post-transcriptionally, miR-328-3p directly restrains CPT1A; loss of this restraint increases fatty-acid oxidation, stem-like behavior and metastatic capacity (10).

Therapy can superimpose additional regulatory inputs. In tamoxifen-resistant ER-positive cells, phosphorylated c-Jun occupies the CPT1A promoter, recruits CBP/p300 and increases H3K27 acetylation, CPT1A transcription and fatty-acid oxidation (16). FDXR depletion likewise reduces CPT1A expression and palmitate-supported respiration, whereas CPT1A re-expression partly restores respiration and growth (17). Radiation-selected breast cancer cells show a related but broader increase in CPT1A/CPT2-dependent fatty-acid oxidation (12). These observations place CPT1A within treatment-responsive metabolic programs, but they do not imply that the same upstream regulator operates in every subtype.

CPT1A regulation should also be separated from substrate supply. PML–PPAR signaling can establish a pro-survival FAO program in breast cancer, whereas adipocyte lipolysis and cancer-associated adipocytes increase the extracellular fatty-acid pool available to tumor cells (74, 81–83). These observations do not show that adipose exposure necessarily raises CPT1A activity; they show that the value of the carnitine shuttle changes when substrate availability changes. Similarly, carnitine abundance, acyl-CoA chain length, CPT2 capacity and downstream β-oxidation can become limiting even when CPT1A protein is abundant. A rigorous regulatory model must therefore distinguish transcription, acute malonyl-CoA control and actual mitochondrial flux.

### 3.3 Beyond ATP production: noncanonical functions of CPT1A

“Beyond ATP production” covers two different propositions. The first concerns non-bioenergetic consequences of conventional fatty-acid oxidation. FAO-derived acetyl-CoA can enter biosynthetic and chromatin-regulatory reactions, and changes in mitochondrial substrate use can reshape redox metabolism. In breast cancer, fatty-acid partitioning has been linked to acetyl-CoA-dependent histone acetylation during epithelial–mesenchymal state transitions (20). This establishes a consequence of FAO, not a direct chromatin function of CPT1A. Nor does β-oxidation directly produce reduced nicotinamide adenine dinucleotide phosphate (NADPH). Any support for glutathione-dependent peroxide control is indirect and depends on the wider metabolic network, including AMPK-regulated preservation of NADPH during stress (29).

The second proposition is that CPT1A may have functions not reducible to carnitine acyltransferase activity. CPT1A has been reported to possess lysine succinyltransferase activity, with enolase 1 identified as a candidate substrate (18). Genetic loss of CPT1 has also produced mitochondrial and lipid phenotypes that were not reproduced by partial pharmacological suppression of β-oxidation (19). These findings raise credible questions about protein acylation, membrane organization and mitochondrial architecture. None has yet been shown to control breast cancer stemness or ferroptosis directly; they should remain mechanistic possibilities, not components inserted into a completed pathway.

Claims of a noncanonical CPT1A function demand a particularly strict experimental design. A phenotype that persists after suppression of FAO may reflect incomplete pathway inhibition, compensatory use of another substrate or a genuine catalytic-independent function. These possibilities can be separated only by combining CPT1A-null cells with wild-type, catalytically inactive and localization-defective rescue constructs. Cross-cancer work showing that NANOG can reprogram both oxidative phosphorylation and fatty-acid metabolism illustrates how a transcriptional stemness program can change the apparent consequence of mitochondrial lipid use (75). In breast cancer, the PML–FAO axis provides a related precedent (74), but neither study establishes that CPT1A itself acts as a transcriptional or epigenetic enzyme. This boundary should remain explicit.

### 3.4 CPT1A dependence is context-specific rather than universal

The available evidence does not support a uniform requirement for CPT1A across breast cancer. In a subset of luminal tumors, CPT1A amplification or overexpression accompanies dependence on fatty-acid oxidation (11). MYC-overexpressing triple-negative breast cancers can also use FAO to meet bioenergetic demand, although the decisive CPT1 isoform was not established in that study (21). Radiation-derived stem-like cells provide a third pattern, in which CPT1A/CPT2 dependence is acquired under therapeutic selection rather than constitutively present (12).

Other observations point in the opposite direction. Across breast cancer cohorts, an FAO-associated expression program has not uniformly predicted adverse outcome, and forced CPT1A expression reduced proliferation and migration in MDA-MB-231 cells in one study (22). This does not negate tumor-promoting evidence. It shows that increased mitochondrial fatty-acid entry may be advantageous in a cell already adapted to FAO, yet disruptive where it increases oxidative load, removes lipids needed for membrane synthesis, or conflicts with an established metabolic program.

CPT1A dependence should consequently be defined functionally, not inferred from expression alone. Convincing studies require genetic perturbation and rescue together with direct flux measurements, ideally isotope-resolved fatty-acid tracing, acylcarnitine profiling and substrate-supported respiration. Pharmacological data require particular caution: etomoxir at concentrations widely used in cancer experiments can alter mitochondrial respiration and CoA homeostasis independently of CPT1 inhibition (19, 23). CPT1A is therefore unlikely to be a universal breast cancer target, but it may become a critical liability in defined subtypes and therapy-selected states.

## 4 CPT1A and the metabolic maintenance of breast cancer stemness

The proposition that CPT1A sustains breast cancer stemness is attractive because it connects a defined mitochondrial transport step with several properties of residual disease: survival during nutrient fluctuation, recovery after treatment, and the capacity to re-enter growth at a distant site. The evidence, however, is not uniform. Some studies manipulate CPT1A directly and recover a stemness phenotype; others establish a requirement for fatty acid oxidation (FAO) without resolving the responsible CPT1 isoform; still others describe oxidative metabolism in breast cancer stem-like cells without testing fatty acids at all. These distinctions are biologically important. They determine whether CPT1A should be viewed as an established dependency in a particular model, a marker of a broader oxidative program, or a plausible but untested point of intervention.

### 4.1 Metabolic features of breast cancer stem cells

The early cancer stem-cell model implied a relatively stable population with a characteristic phenotype. Breast tumors do not behave so neatly. CD44high/CD24low and ALDHhigh cells are enriched for tumor-initiating activity, but they are neither equivalent nor fixed. The former population tends to occupy a relatively quiescent, mesenchymal-like state, whereas ALDHhigh cells more often display an epithelial, proliferative phenotype. Lineage and state-tracing experiments have shown that the two compartments can interconvert and that their spatial distribution within tumors differs (5). Metabolism should be interpreted against this background. A metabolic profile measured in one marker-defined fraction is not automatically a universal property of all BCSCs, and a treatment that depletes one fraction may leave the capacity to regenerate it intact.

The apparently conflicting literature on glucose and mitochondrial metabolism illustrates the point. Using mammosphere cultures and a proteomic-metabolomic approach, Ciavardelli and colleagues found enrichment of glycolytic enzymes, increased fermentative metabolism, and sensitivity to 2-deoxyglucose in stem-like breast cancer cells (25). Hypoxia can reinforce such a program: PDK1 induction diverts pyruvate away from mitochondrial oxidation, increases glycolytic output, and supports ALDH positivity, sphere formation, and tumor initiation (26). These observations are internally coherent, particularly for cells growing in poorly perfused regions where oxygen availability constrains respiration and glycolysis supplies both ATP and biosynthetic intermediates.

Other models point in the opposite direction. Vlashi and colleagues reported that mammosphere-enriched BCSCs generated less lactate, contained more ATP, and exhibited greater maximal respiratory capacity than differentiated progeny across luminal, basal, and claudin-low cell lines (24). Radiation further increased this energetic phenotype. In TNBC, MYC and mitochondrial MCL1 were subsequently shown to cooperate in raising oxidative phosphorylation, reactive oxygen species (ROS), HIF-1α activity, and the abundance of chemotherapy-resistant tumor-initiating cells (27). These studies do not invalidate the glycolytic data. They show that the experimental definition of stemness, the proliferative state of the cells, and the stress used to enrich them all influence the pathway that appears dominant.

Redox state provides a more useful organizing principle than fuel preference alone. Mesenchymal-like BCSCs are relatively glycolytic and maintain lower ROS, whereas epithelial-like BCSCs rely more strongly on mitochondrial oxidative metabolism and compensate with NRF2-dependent thioredoxin and glutathione systems (6). The two states respond differently to glycolytic and antioxidant interventions, yet remain connected by reversible transitions. Thus, oxidative metabolism can be both supportive and hazardous: it supplies energy and biosynthetic precursors but also raises the burden of electron leakage and peroxide detoxification. A BCSC that increases mitochondrial activity must acquire a proportionate redox-buffering capacity or leave the stem-like state.

FAO fits this model as a conditional component of metabolic plasticity rather than a defining feature of every BCSC. JAK/STAT3-driven FAO supports self-renewal and chemoresistance in several breast cancer models, particularly when adipocyte-derived leptin is available (7). Importantly, CPT1B was the principal STAT3 target in that study; it should not be cited as direct evidence for CPT1A. By contrast, the miR-328-3p–CPT1A study and more recent work on OVOL2-deficient TNBC provide direct evidence that CPT1A-containing FAO programs can support stem-like phenotypes (10, 28). Taken together, these data suggest that breast cancer cells recruit different CPT1 isoforms according to lineage and microenvironmental context. The common biological requirement is access to mitochondrial fatty-acid catabolism when glucose, oxygen, or therapy changes the relative value of available substrates.

This distinction also explains why BCSC metabolism is more accurately described as flexible than simply glycolytic or oxidative. A stem-like cell in a hypoxic primary-tumor niche may favor glycolysis; the same clone, after detachment or treatment, may depend on mitochondrial oxidation to preserve ATP and redox balance. At metastatic sites, local adipocytes and stromal cells can alter both lipid supply and the signals that control fatty-acid use. What is selected over the course of disease is therefore not a single pathway, but the ability to move between pathways without losing tumor-initiating competence. CPT1A becomes relevant where that flexibility specifically requires the mitochondrial entry of long-chain fatty acids.

The history of BCSC markers explains why apparently similar studies can reach different metabolic conclusions. CD44high/CD24low cells were first isolated by tumor-initiation assays, whereas ALDH1 identified an overlapping epithelial-like compartment with prognostic value (62, 63). These populations can regenerate one another through stochastic and epigenetically poised transitions (64–66), and EMT-associated stemness is distributed across partial as well as fully mesenchymal states (67–70). Early metastatic breast cancer cells likewise carry a stem-like program that is not maintained uniformly during outgrowth (71). Metabolic experiments should therefore report which state was studied, whether state conversion occurred during treatment, and whether the phenotype was confirmed by serial functional assays. Without this information, a change in ALDH activity or mammosphere number cannot distinguish selective killing from altered proliferation or state redistribution.

### 4.2 Direct evidence linking CPT1A to breast cancer stemness

The most direct breast cancer evidence comes from studies in which CPT1A is genetically manipulated and stemness is assessed functionally. Zeng and colleagues identified CPT1A as a direct target of miR-328-3p and traced the consequences through fatty-acid oxidation, stem-like behavior, and metastasis (10). Increasing miR-328-3p reduced CPT1A expression, FAO-related readouts, ALDH-associated stemness and sphere formation, whereas CPT1A re-expression restored key phenotypes. The study also linked lower miR-328-3p and higher CPT1A to metastatic disease and demonstrated effects on experimental metastasis. Its strength lies in the target-and-rescue design. Its limitations are equally clear: mammosphere and marker assays provide enrichment rather than an exclusive definition of BCSCs, and the biochemical fate of individual fatty-acid species was not resolved.

In hormone receptor-positive disease, Jariwala and colleagues approached CPT1A from a different direction (11). Proteogenomic analyses placed CPT1A within the recurrent 11q13–14 amplicon and associated its abundance with highly proliferative luminal tumors and adverse outcome. CPT1A depletion reduced proliferation, survival, colony formation, and mammosphere formation, whereas ectopic expression increased these properties. FAO inhibition also suppressed orthotopic tumor growth. This work establishes that CPT1A is not merely a marker carried by the amplicon; it contributes to the fitness of at least a subset of luminal tumors. Nevertheless, increased mammosphere formation in an otherwise proliferative model does not by itself demonstrate that CPT1A selectively controls a self-renewing compartment. Limiting-dilution transplantation, serial passage, and lineage-resolved experiments would strengthen that conclusion.

Radioresistant models provide a third line of direct evidence. Han and colleagues found increased CPT1A and CPT2 expression, enhanced FAO, and greater mitochondrial energy production in radioresistant breast cancer cells and radiation-derived BCSCs (12). Pharmacological FAO inhibition and CRISPR-mediated disruption of CPT1A or CPT2 reduced aggressive growth and radioresistance, with accompanying suppression of radiation-induced ERK signaling. The use of both genetic and metabolic readouts is important, because it connects carnitine-shuttle activity to a therapy-selected stem-like population rather than inferring function from expression alone. The model remains treatment-specific, and CPT1A and CPT2 were perturbed as parts of the same shuttle. It therefore establishes dependence on CPT1A-mediated mitochondrial import more securely than it establishes a unique, CPT2-independent function of CPT1A.

Recent work in OVOL2-deficient TNBC substantially strengthens the causal chain. Loss of the epithelial transcription factor OVOL2 activated JAK/STAT3 signaling, increased transcription of both CPT1A and CPT1B, raised palmitate-supported oxygen consumption and 13C-palmitate incorporation, and increased ATP and NADPH availability (28). These changes were accompanied by greater mammosphere formation, tumor initiation, serial transplantability, and metastasis. STAT3 manipulation and pharmacological inhibition of JAK or FAO reversed major components of the phenotype, including in an MMTV-PyVT/Ovol2 model. This is compelling evidence that a CPT1-containing FAO program sustains stemness when OVOL2 restraint is lost. It is not, however, evidence that CPT1A alone accounts for the phenotype, because CPT1B is co-regulated and isoform-specific rescue was not the central experimental design.

Several influential studies support the broader FAO–stemness relationship but require similar restraint in attribution. Wang and colleagues demonstrated that adipocyte-derived leptin activates STAT3, induces CPT1B, and drives FAO-dependent BCSC self-renewal and chemoresistance (7). Loo and colleagues showed that FAO controls epithelial–mesenchymal cell-state transitions, tumorigenicity, and metastatic behavior through changes in fatty-acid partitioning and acetyl-CoA-dependent histone acetylation (20). Camarda and colleagues identified FAO as a metabolic vulnerability in MYC-overexpressing TNBC (21). Each study makes the CPT1A hypothesis more plausible; none should be converted into direct CPT1A evidence when the tested node was CPT1B, aggregate CPT1 activity, or the pathway as a whole.

The present evidence therefore supports a graded conclusion. CPT1A has direct causal support in miR-328-3p-regulated breast cancer, CPT1A-amplified luminal disease, and radiation-selected models (10–12). A combined CPT1A/CPT1B program is strongly implicated in OVOL2-deficient TNBC (28). Other studies demonstrate that FAO can maintain stemness without identifying CPT1A as the decisive isoform (7, 20, 21). **Table 2** summarizes these relationships and assigns the evidence level to the specific link being claimed, rather than to the publication as a whole (**Table 2**).

**Table 2:** Evidence linking CPT1A and fatty-acid oxidation to breast cancer stemness and therapy persistence.

Study/context	Metabolic node and intervention	Functional metabolic evidence	Stemness or persistence endpoint	Evidence appraisal
Zeng et al. (10); metastatic breast cancer models	miR-328-3p targeting of CPT1A; CPT1A rescue; FAO inhibition	FAO-related readouts and CPT1A-dependent rescue	ALDH-associated phenotype, spheroids, invasion and metastasis	Level I for the miR-328-3p–CPT1A–FAO link; functional BCSC assays present
Jariwala et al. (11); HR-positive luminal breast cancer	CPT1A knockdown/overexpression and pharmacological FAO inhibition	CPT1A/FAO-associated growth and survival	Mammosphere and colony formation; orthotopic growth	Level I for CPT1A-dependent tumor fitness; Level II for selective BCSC dependence
Han et al. (12); radioresistant cells and radiation-derived BCSCs	CRISPR disruption of CPT1A or CPT2; FAO inhibition	FAO and mitochondrial energy metabolism	Aggressive growth, clonogenic survival and radioresistance	Level I for carnitine-shuttle dependence in radiation-selected stem-like cells
Lu et al. (28); OVOL2-deficient TNBC	OVOL2–JAK/STAT3 control of CPT1A and CPT1B; JAK/FAO inhibition	Palmitate-supported OCR, 13C-palmitate tracing, ATP and redox readouts	Mammospheres, serial tumor initiation and metastasis	Level I for a combined CPT1A/CPT1B FAO program; not CPT1A-specific
Wang et al. (7); adipocyte-supported BCSCs	Leptin–STAT3–CPT1B signaling and FAO inhibition	FAO activity downstream of CPT1B	Self-renewal, limiting dilution and chemoresistance	Level I for CPT1B–FAO; not direct CPT1A evidence
Loo et al. (20); basal-like plasticity models	FAO perturbation and retinoid-induced lipid repartitioning	Fatty-acid flux, acetyl-CoA and histone acetylation	EMT/MET, tumorigenicity and metastasis	Level I for pathway-level FAO control; CPT1A isoform not resolved
Jiang et al. (16) and Yan et al. (17); endocrine-resistant ER-positive disease	c-Jun or FDXR regulation of CPT1A	CPT1A-dependent FAO and mitochondrial adaptation	Tamoxifen/endocrine resistance and tumor growth	Level I for CPT1A in endocrine resistance; BCSC identity incompletely tested
Ahn et al. (31); endocrine-resistant ER-positive disease	CPT1 suppression, FAO inhibitors and Src targeting	Integrated metabolomics/lipidomics; AMPK–FAO–OXPHOS activity	Endocrine resistance *in vitro* and *in vivo*	Level I for CPT1/FAO dependence; isoform and BCSC specificity unresolved

### 4.3 CPT1A-mediated FAO supports stemness through energy and redox homeostasis

The simplest explanation for the association between CPT1A and stemness is energetic. Long-chain fatty acids are energy-dense substrates, and their oxidation can sustain mitochondrial ATP production when glucose availability falls or when treatment increases the cost of macromolecular repair. In radioresistant cells, CPT1A/CPT2 upregulation was accompanied by increased FAO and mitochondrial energy metabolism; interfering with the shuttle reduced survival after irradiation (12). OVOL2-deficient TNBC cells likewise used more palmitate, generated more ATP, and displayed enhanced tumor-initiating activity (28). These data support an energy-survival link, but ATP alone is unlikely to explain stemness. Many rapidly proliferating non-stem cells have high ATP demand, and restoration of ATP does not necessarily recreate a stem-cell state.

The value of FAO is greatest under conditions in which the competing carbon pathways are constrained. During glucose limitation, detachment, or therapy, AMPK inhibits ACC1 and ACC2, lowering malonyl-CoA and relieving inhibition of CPT1. This response both limits ATP-consuming lipid synthesis and permits greater mitochondrial fatty-acid entry. Work across tumor models has shown that the AMPK–ACC axis preserves survival during energy stress by coordinating ATP production and NADPH homeostasis (29). The mechanistic language requires care: β-oxidation produces NADH and FADH2 directly, not cytosolic NADPH. NADPH can nevertheless be maintained indirectly through reduced consumption by fatty-acid synthesis and through mitochondrial and cytosolic reactions coupled to citrate, malate, isocitrate, and one-carbon metabolism. The net effect depends on the surrounding metabolic network.

This indirect redox contribution is relevant to BCSCs because a low or tightly buffered ROS state supports self-renewal in some compartments, whereas excessive ROS promotes differentiation or death. In OVOL2-deficient cells, increased FAO coincided with higher NADPH/NADP+ and reduced-to-oxidized glutathione (GSH/GSSG) ratios and lower ROS; acetate or N-acetylcysteine partly rescued the effects of OVOL2 restoration on mammosphere formation and tumor initiation (28). In the redox-state model developed by Luo and colleagues, epithelial BCSCs were dependent on NRF2-regulated thioredoxin and glutathione pathways, whereas mesenchymal BCSCs used a distinct glycolytic-redox program (6). CPT1A-mediated FAO may therefore support stemness not by imposing uniformly low ROS, but by expanding the range of oxidative flux that a cell can tolerate without losing self-renewal capacity.

FAO also changes the availability of acetyl-CoA. Mitochondrial acetyl-CoA cannot cross the inner membrane directly, but citrate export and ATP-citrate lyase can transfer carbon into the nucleo-cytosolic acetyl-CoA pool. In breast cancer cell-state models, FAO-dependent acetyl-CoA influenced histone acetylation at EMT-associated genes and thereby contributed to maintenance of a mesenchymal program (20). This offers a route by which substrate use can become transcriptional memory: a transient increase in FAO may alter chromatin in ways that persist beyond the immediate energetic need. The magnitude and direction of that effect will depend on citrate export, ATP-citrate lyase activity, acetate utilization, and competing demands for acetyl-CoA. It should not be assumed that every increase in CPT1A produces global histone hyperacetylation.

Mitochondrial fitness is a further component of the phenotype. FAO requires an intact electron-transport chain, appropriate cofactor balance, and the capacity to manage ROS. Conversely, sustained fatty-acid entry can reinforce mitochondrial biogenesis and substrate flexibility through AMPK, PGC-1α, and stress-responsive transcription. Chemotherapy-resistant TNBC illustrates how a mitochondrial program can enrich tumor-initiating cells: MYC and mitochondrial MCL1 cooperate to increase oxidative phosphorylation and ROS-dependent HIF-1α signaling (27). CPT1A was not tested as the driver in that study, so it should be used as evidence that therapy-resistant BCSCs can depend on mitochondrial respiration, not as proof of a CPT1A–MYC–MCL1 pathway.

There is also a ceiling to the benefit of FAO. Greater electron flux can increase superoxide generation, and incomplete oxidation may produce acylcarnitine accumulation and metabolic stress. High CPT1A expression is therefore advantageous only when electron transport, antioxidant defense, and lipid supply remain coordinated. This helps explain why CPT1A dependence varies among subtypes and treatment states. In one context, CPT1A provides a reserve fuel and reduces redox stress; in another, the same pathway may increase oxidative burden or be bypassed by glycolysis, glutamine oxidation, or acetate use. A rigorous claim that CPT1A maintains stemness through energy and redox homeostasis should consequently include FAO flux, ATP and redox measurements, functional stemness assays, and rescue experiments that separate energy failure from nonspecific toxicity.

Energy yield alone is not sufficient to explain why FAO supports stemness. β-oxidation produces NADH and FADH2, not NADPH, and its contribution to peroxide control depends on how mitochondrial carbon is coupled to citrate export, malic enzyme, isocitrate dehydrogenase and pentose-phosphate activity. AMPK can preserve NADPH during energy stress by suppressing fatty-acid synthesis (29), while PML-dependent FAO provides a pro-survival program in breast cancer (74). In tumor-initiating cells from another cancer context, NANOG-driven metabolic reprogramming lowered oxidative stress while redirecting fatty-acid use (75). These observations support a network model: CPT1A may secure substrate access, but the protective phenotype emerges only when electron transfer, NADPH regeneration and glutathione or thioredoxin systems remain coordinated.

### 4.4 CPT1A, EMT, and phenotypic plasticity

EMT is often used as a surrogate for stemness, but the relationship is not one-to-one. A fully mesenchymal state may favor motility and resistance to detachment while compromising proliferative outgrowth; metastatic colonization frequently requires at least partial epithelial reversion. The two major BCSC phenotypes mirror this division of labor. CD44high/CD24low cells are enriched for mesenchymal and invasive properties, whereas ALDHhigh cells are more epithelial and proliferative (5). Cells can move between these states, which means that the relevant therapeutic target may be the transition itself rather than either endpoint.

Lipid metabolism participates directly in this transition. In basal-like breast cancer models, Loo and colleagues found that mesenchymal cells preferentially directed fatty acids toward β-oxidation, whereas retinoid-induced mesenchymal-to-epithelial transition redirected the same substrates toward neutral-lipid storage (20). Perturbing FAO promoted a more epithelial phenotype and impaired EMT-driven metastasis. Mechanistically, FAO-derived acetyl-CoA contributed to histone acetylation at EMT-associated loci. The importance of this work is not simply that mesenchymal cells “use more fat.” It shows that fatty-acid fate can help stabilize a transcriptional state and that lipid storage and oxidation are dynamically coupled to plasticity.

An independent TGF-β1 model reached a compatible conclusion: EMT in MCF7 cells was accompanied by AMPK activation, reduced ACC activity, increased FAO and oxidative phosphorylation, and higher CPT1 expression in mesenchymal-like cells (30). This study did not resolve CPT1A-specific causality or functional BCSC frequency, but it supports the metabolic direction observed by Loo and colleagues. Together, the findings place the AMPK–ACC–CPT1 axis within EMT-associated metabolic reprogramming while leaving open which CPT1 isoform is dominant in different lineages.

Direct CPT1A evidence adds two complementary regulatory routes. In the miR-328-3p model, release of CPT1A from microRNA repression increased FAO, stem-like characteristics, invasion, and metastatic capacity (10). In OVOL2-deficient TNBC, activation of JAK/STAT3 induced both CPT1A and CPT1B, increased FAO, and enhanced tumor initiation and lung metastasis (28). OVOL2 is itself an epithelial-state regulator, making this study especially relevant to the intersection of transcriptional plasticity and metabolism. Yet its interpretation should remain isoform-aware: the phenotype reflects a CPT1A/CPT1B program downstream of altered epithelial identity, not a demonstration that CPT1A alone initiates EMT.

The direction of causality is likely bidirectional. EMT-associated signals can activate FAO through AMPK, STAT3, and changes in malonyl-CoA; FAO can then reinforce the mesenchymal program through energy supply, redox adaptation, and acetyl-CoA-dependent chromatin regulation. Such feedback would be advantageous during invasion, when cells face matrix detachment and irregular nutrient delivery. It may become disadvantageous at the metastatic site if continued mesenchymal commitment prevents proliferative colonization. Repartitioning fatty acids toward storage during epithelial reversion could therefore be part of the transition from dissemination to outgrowth rather than a simple loss of metabolic fitness.

This interpretation avoids two common overstatements. First, EMT markers do not establish tumor-initiating capacity; functional transplantation remains necessary. Second, an FAO-high mesenchymal state should not be equated with a permanently FAO-dependent BCSC. The clinically difficult property is reversible state switching. CPT1A may support one phase of that cycle, particularly in cells using long-chain fatty acids during invasion or treatment stress, but the same cells may later adopt glycolytic or lipid-storing programs. Therapeutic inhibition of CPT1A will be most effective when applied at a state transition that cannot be readily bypassed by another substrate pathway.

EMT is similarly better viewed as a landscape than an endpoint. Poised ZEB1 chromatin permits rapid acquisition of stem-like features (66), and lineage-resolved studies identify several partial EMT states with distinct metastatic capacities (69, 70). This matters for ferroptosis because mesenchymal and drug-tolerant states can become unusually dependent on GPX4 even while retaining strong antioxidant defenses (55). A plausible interpretation is that epithelial–mesenchymal plasticity simultaneously increases exposure to oxidizable membrane lipids and selects for compensatory peroxide detoxification. Whether CPT1A shifts during that transition, and whether it changes lipid composition or simply supplies energy for the transition, must be tested rather than inferred from a common association with aggressiveness.

### 4.5 CPT1A in therapy-induced stem-like states

Treatment can enrich stem-like cells by at least two routes. It may select a pre-existing population that already tolerates stress, or it may induce non-stem cells to enter a reversible drug-tolerant state. Most experimental systems cannot fully separate these possibilities, and the distinction matters. Selection implies that the metabolic program is present before therapy and could serve as a predictive biomarker; induction implies that timing and sequence may be more important than baseline expression. The available CPT1A data support both pre-existing dependence and treatment-driven adaptation, but the balance differs by treatment modality.

The radiotherapy evidence is the most direct. Radiation-derived BCSCs and chronically radioresistant breast cancer cells increase CPT1A/CPT2 expression and FAO, consistent with the earlier observation that irradiation heightens the energetic state of mammosphere-enriched BCSCs (12, 24). Disrupting CPT1A or CPT2, or inhibiting FAO pharmacologically, impaired ERK activation, clonogenic growth, and radioresistance (12). A plausible interpretation is that radiation raises the need for ATP-dependent repair and antioxidant buffering, selecting cells able to mobilize mitochondrial lipid oxidation. However, the study did not show that CPT1A inhibition specifically eliminated all tumor-reinitiating cells *in vivo*. The safer conclusion is that the carnitine shuttle supports a radiation-selected stem-like state and represents a rational radiosensitization target.

Endocrine therapy produces a related but molecularly distinct adaptation. In tamoxifen-resistant ER-positive cells, phosphorylated c-Jun binds the CPT1A promoter and recruits CBP/P300, increasing H3K27 acetylation, CPT1A transcription, FAO, and resistance (16). FDXR has also been linked to primary and endocrine-resistant ER-positive tumor growth through CPT1A-mediated FAO (17). Multi-omics analyses of endocrine-resistant models identified an AMPK–FAO–OXPHOS program in which CPT1 suppression or FAO inhibition restored sensitivity to endocrine therapy; increased FAO also activated Src signaling (31). These studies establish a recurrent CPT1A/FAO dependency in endocrine resistance. They do not yet demonstrate that the resistant cells are functionally defined BCSCs, even though long-term persistence, mammosphere capacity in luminal models, and late recurrence make the connection biologically plausible (11).

Chemotherapy adds another layer. The best BCSC-specific FAO evidence in this setting is the adipocyte–leptin–STAT3–CPT1B pathway described by Wang and colleagues (7). Blocking FAO or leptin signaling reduced self-renewal and resensitized tumors to chemotherapy. This is strong evidence that lipid-rich microenvironments can sustain chemoresistant BCSCs, but the isoform was CPT1B, not CPT1A. In TNBC, chemotherapy can also select MYC/MCL1-driven cells with high oxidative phosphorylation and enhanced tumor-initiating activity (27). Again, the finding supports mitochondrial dependence rather than CPT1A-specific causality. Direct evidence that CPT1A is the indispensable FAO isoform in chemotherapy-persistent BCSCs remains limited, despite the broader association of CPT1A with metastasis and stemness (10).

The same caution applies to targeted therapy. In HER2-positive breast cancer, PPARG activation has been linked to fatty-acid metabolic reprogramming and resistance to trastuzumab, lapatinib, and pyrotinib (32). This makes lipid metabolism a credible component of targeted-therapy persistence, but it does not establish either FAO dependence or a CPT1A-dependent BCSC mechanism. HER2-positive disease is therefore an important evidence gap rather than a completed part of the CPT1A narrative. Future studies should determine whether anti-HER2 therapy induces a CPT1A-high residual population, whether that population has serial tumor-initiating capacity, and whether isoform-specific CPT1A suppression prevents regrowth.

Across treatment classes, several principles emerge. First, a rise in CPT1A expression after therapy is not sufficient evidence of FAO dependence; palmitate tracing, acylcarnitine measurements, and substrate-resolved respiration are required. Second, a reduction in mammosphere formation after treatment may reflect cytotoxicity or reduced proliferation rather than loss of self-renewal. Third, pharmacological data obtained with etomoxir or perhexiline should be paired with genetic suppression and rescue because both compounds have effects beyond CPT1A at commonly used concentrations (19, 23). Finally, timing should be treated as a mechanistic variable. Pretreatment may prevent metabolic adaptation, whereas post-treatment inhibition may be better suited to eliminating an already selected residual population.

The working model is thus narrower, but more defensible, than the claim that all therapy-resistant BCSCs are CPT1A dependent. Radiotherapy generates the clearest CPT1A/CPT2-linked stem-like state; endocrine therapy repeatedly selects CPT1A/FAO-dependent survival programs without yet resolving their BCSC identity; chemotherapy provides strong FAO–BCSC evidence that is often CPT1B- or OXPHOS-centered; and anti-HER2 therapy remains largely inferential. **Figure 2** integrates these relationships while distinguishing direct breast cancer evidence from pathway-level or hypothesis-generating connections (**Figure 2**).

**Figure 2:**
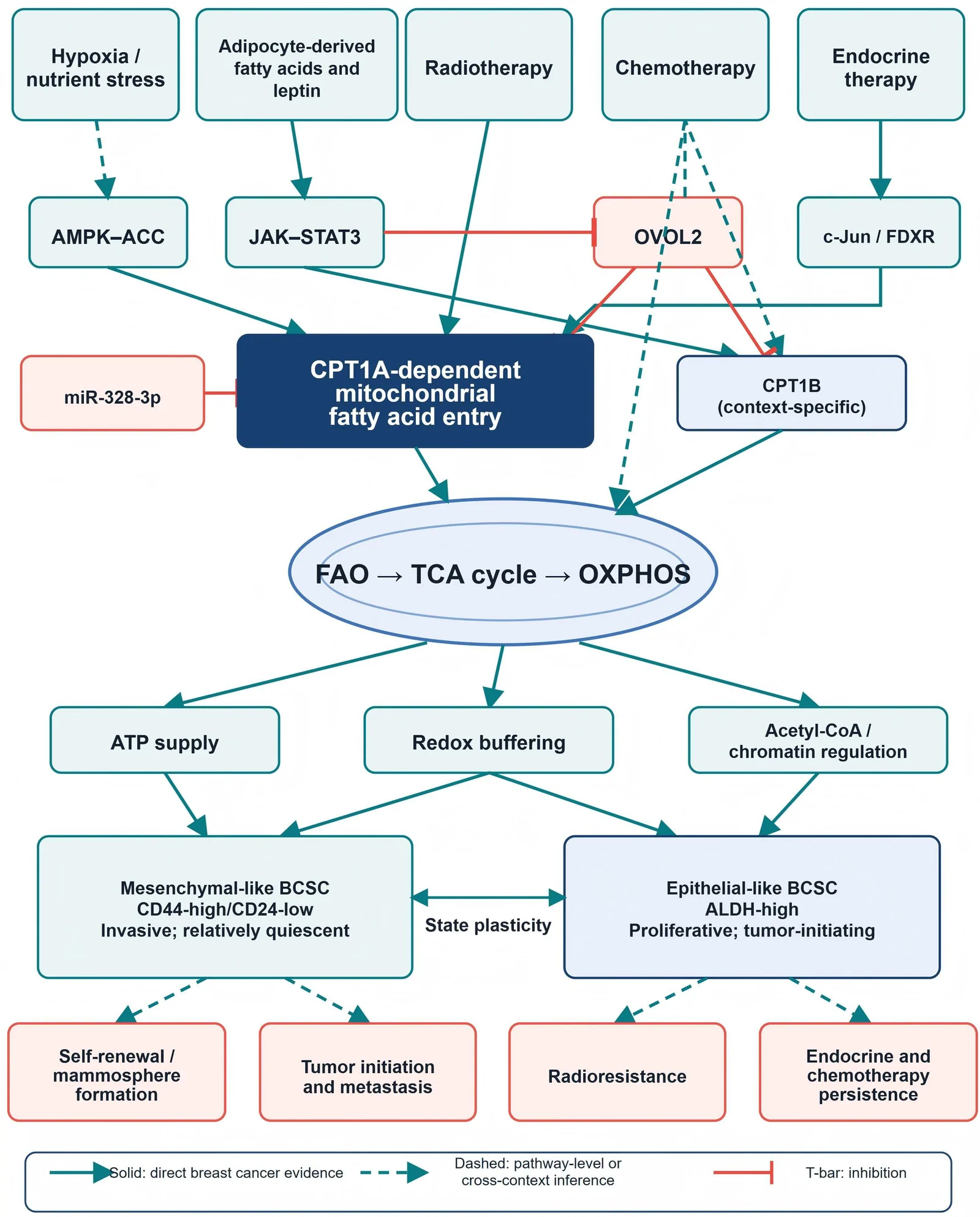
CPT1A-driven metabolic plasticity sustains breast cancer stemness and therapy persistence. Environmental and therapeutic pressures recruit distinct regulatory routes to CPT1A or, in adipocyte-supported and some chemotherapy contexts, CPT1B. CPT1-dependent FAO and oxidative phosphorylation support ATP supply, redox buffering and acetyl-CoA-linked chromatin regulation across interconvertible mesenchymal-like and epithelial-like BCSC states. Solid arrows denote direct breast cancer evidence; dashed arrows denote pathway-level or cross-context inference; T-bars denote inhibition.

Therapy-induced stemness need not arise only through selection of a pre-existing rare clone. Residual breast tumors undergo measurable metabolic shifts that can drive recurrence (73), and endocrine-resistant metastatic disease contains self-renewing CD133high cells maintained by IL-6–Notch3 signaling (80). Disseminated cells are further shaped by perivascular dormancy signals, WNT-restrained latency, primary-tumor hypoxia and, in HER2-driven models, dissemination before overt tumor formation (76–79). In parallel, therapy-tolerant persister states across cancer models acquire a marked dependence on GPX4 (55, 56). These findings support a temporal model in which treatment first narrows the population, then stromal and metabolic cues stabilize a residual state. CPT1A may be essential during only part of this sequence, which is why intervention timing should be studied as carefully as target expression.

## 5 Ferroptosis vulnerability and resistance in breast cancer stem cells

### 5.1 The ferroptosis machinery: a concise overview

Ferroptosis is initiated when the rate of phospholipid peroxide formation exceeds the capacity of membrane-protective systems (33). Its defining substrate is not total cellular fat, but a restricted pool of oxidizable phospholipids. ACSL4 activates arachidonic and adrenic acids, and LPCAT3 incorporates these acyl chains into membrane phospholipids; oxidation of the resulting phosphatidylethanolamine species produces signals closely associated with ferroptotic death (35, 36). Iron promotes radical propagation directly and supports iron-dependent enzymes that contribute to lipid oxidation. The pathway is therefore governed by three variables that cannot be inferred from one another: the labile iron pool, the abundance and location of susceptible phospholipids, and the capacity to reduce lipid hydroperoxides.

The system xc−–GSH–GPX4 axis is the best-characterized protective pathway. SLC7A11 imports cystine in exchange for glutamate, cysteine derived from cystine supports glutathione synthesis, and GPX4 uses glutathione to reduce phospholipid hydroperoxides (33, 34). Cells can nevertheless resist ferroptosis when this axis is weakened. Ferroptosis suppressor protein 1 (FSP1) regenerates reduced coenzyme Q at non-mitochondrial membranes (37, 38); mitochondrial dihydroorotate dehydrogenase (DHODH) provides a parallel CoQH2-based defense (39); and the GTP cyclohydrolase 1–tetrahydrobiopterin (GCH1–BH4) pathway changes the antioxidant and phospholipid landscape independently of GPX4 (40). MUFAs can also displace oxidizable PUFAs from phospholipids through ACSL3-dependent remodeling (41). These systems are partly redundant, which explains why GPX4 abundance alone is an incomplete predictor of ferroptosis sensitivity.

For this review, two points are particularly relevant. First, CPT1A sits upstream of substrate allocation rather than within the canonical ferroptosis machinery. Its effect is therefore expected to depend on which fatty acids are available and whether they would otherwise enter neutral lipids or PUFA-containing phospholipids. Second, mitochondrial metabolism has opposing effects: it can support antioxidant defense, but electron transport can also add oxidative pressure. The direction of the CPT1A–ferroptosis relationship must be measured in each cellular state; it should not be inferred from the textbook function of FAO.

Ferroptosis is not equivalent to generalized oxidative stress. Its execution depends on the oxidation of defined phospholipids and on failure of the systems that reduce phospholipid hydroperoxides. GPX4 is the central glutathione-dependent enzyme in this process (46). PEBP1 can organize lipoxygenase-dependent production of death-associated phospholipid signals, ether phospholipids can either increase susceptibility or be remodeled during evasion, and cytochrome P450 oxidoreductase contributes to phospholipid peroxidation (47–49). Mitochondria are important in cysteine-deprivation-induced ferroptosis but are less uniformly required when GPX4 is directly inhibited (50). Tumor-suppressive inputs, including p53-dependent repression of SLC7A11, add another layer (51). The relevant endpoint is therefore oxidized membrane phospholipid burden, not total cellular ROS.

Antiferroptotic defense is likewise distributed across several compartments. Selenium utilization and uninterrupted selenoprotein translation are required to maintain functional GPX4 (91, 92). FSP1, DHODH and the GCH1–tetrahydrobiopterin system provide partially parallel protection (37–40), while a non-canonical vitamin K cycle can suppress ferroptosis independently of the canonical GPX4 pathway (52). Cells may also lower the labile iron pool: prominin-2 promotes ferritin-containing multivesicular bodies and iron export after ferroptotic stress (53). This redundancy explains why GPX4 abundance alone is an unreliable measure of ferroptosis resistance. A BCSC may compensate for modest GPX4 loss through CoQ reduction, membrane remodeling or iron handling, and the dominant defense may change after treatment.

### 5.2 Iron addiction as an Achilles’ heel of cancer stem cells

Stem-like tumor cells often maintain high iron demand for ribonucleotide reduction, mitochondrial iron–sulfur proteins, and epigenetic enzymes. They may also accumulate ferritin and develop a larger reserve that can be mobilized under stress. This creates a paradox. Iron supports tumor initiation and metabolic competence, but the same reserve can accelerate lipid oxidation if antioxidant protection fails. The relevant vulnerability is not simply “high iron,” but dependence on tightly regulated iron traffic.

Breast cancer provides functional evidence for this idea. Inhibition or deletion of the lysosomal channel TRPML1 caused lysosomal iron retention, increased lipid ROS, reduced ALDH-positive and CD44high/CD24low fractions, impaired mammosphere formation, and suppressed tumor growth; liproxstatin-1 and iron chelation rescued key phenotypes (42). The result is notable because it links a defined iron-handling mechanism to both ferroptosis and BCSC function. Salinomycin and its derivative ironomycin provide an independent example: lysosomal iron sequestration, ferritin degradation and iron-dependent ROS preferentially depleted breast cancer stem-like cells and reduced tumor-seeding capacity (45). These observations do not imply that all BCSCs are iron-loaded in the same way, but they show that the lysosome–iron–lipid peroxide axis can be therapeutically exposed.

Hypoxic BCSCs illustrate how the vulnerability can be concealed. Cystathionine β-synthase supports an alternative source of cysteine under hypoxia; its inhibition increased lipid peroxidation and reduced stem-like cells in breast cancer models (43). Thus, BCSCs may protect an iron-dependent state by diversifying cysteine supply rather than by reducing iron demand. Therapeutically, this favors strategies that combine disruption of iron buffering with blockade of peroxide detoxification rather than relying on iron delivery alone.

### 5.3 Direct ferroptosis-resistance mechanisms in breast cancer stem cells

The clearest evidence that ferroptosis resistance can be embedded in a stemness program comes from two complementary studies. Wu and colleagues showed that BCSCs secrete DKK1, which promotes differentiation of neighboring tumor cells and enables metastatic outgrowth (8). DKK1 increased SLC7A11 expression and protected disseminated cells from lipid peroxidation and ferroptosis. Combined DKK1 inhibition and ferroptosis induction reduced metastatic burden more effectively than either intervention alone. In this setting, BCSCs do not simply protect themselves; they remodel the phenotype and redox defense of cells that seed metastases.

Luo and colleagues identified an intracellular program centered on ZMYND8 and NRF2 (9). In ALDHhigh BCSCs, ZMYND8 reduced ROS and iron, silenced KEAP1, stabilized NRF2, and recruited NRF2 to antioxidant genes. Genetic loss of ZMYND8 increased lipid peroxidation and ferroptotic death, depleted ALDHhigh cells, and reduced tumor initiation; ferroptosis inhibitors rescued mammosphere and BCSC phenotypes. NRF2 in turn induced ZMYND8, creating a positive feedback loop. This study meets a high standard for ferroptosis assignment because it combines iron and lipid-peroxidation measurements, pharmacological rescue, genetic intervention, and functional stemness assays.

Additional studies broaden the range of actionable defenses. TRPML1 inhibition triggers ferroptosis in BCSCs by disrupting lysosomal iron handling (42), while inhibition of hypoxia-induced cystathionine β-synthase reduces cysteine availability and exposes hypoxic stem-like cells (43). These mechanisms converge on the same outcome but operate at different levels: iron mobilization, sulfur-amino-acid supply, and transcriptional antioxidant control. **Table 3** summarizes the direct BCSC evidence and separates rigorous ferroptosis validation from studies based primarily on marker changes (**Table 3**).

**Table 3:** Ferroptosis-regulatory mechanisms in breast cancer stem-like cells.

Regulator/study	Mechanism	BCSC or disease model	Ferroptosis validation	Functional implication	Evidence level
DKK1–SLC7A11 (8)	BCSC-secreted DKK1 increases SLC7A11 and limits lipid peroxidation	Breast cancer dissemination and metastatic outgrowth	Lipid peroxidation, ferroptosis modulation and combination rescue	Protects disseminated cells and permits metastatic colonization	I
ZMYND8–NRF2 (9)	KEAP1 silencing, NRF2 stabilization/recruitment and reduced iron	ALDH-high BCSCs; mammospheres and mouse tumors	Iron and lipid-peroxidation measurements; ferroptosis inhibitor rescue	Maintains BCSC expansion and tumor initiation	I
TRPML1 (42)	Lysosomal iron retention and lipid ROS after channel inhibition	HCC1954 BCSCs and xenografts	C11-BODIPY, Fe2+, liproxstatin-1 and DFO rescue	Reduces ALDH-positive/CD44-high cells, spheres and tumor growth	I
Hypoxia-induced CBS (43)	Alternative cysteine supply under hypoxia	TNBC stem-like cells and mouse breast tumors	Lipid peroxidation and ferroptosis-associated rescue/response	Exposes hypoxic BCSCs to ferroptosis	I–II
RACGAP1–PPARα–CPT1A (13)	CPT1A-dependent fatty-acid metabolism limits lipid peroxidation	TNBC cells and tumors	Ferroptosis rescue and CPT1A restoration	Direct CPT1A–ferroptosis link; BCSC specificity not established	I for ferroptosis; II for stemness
Salinomycin/ironomycin (45)	Lysosomal iron sequestration, ferritin degradation and iron-dependent ROS	Breast CSC cultures and patient-derived xenografts	Lysosomal iron and lipid-ROS analyses; death pathway consistent with ferroptosis	Depletes ALDH-positive cells and reduces tumor-seeding capacity	II
CPT1A–c-Myc–NRF2/GPX4 (44)	CPT1A stabilizes c-Myc, activates NRF2/GPX4 and suppresses ACSL4	Lung cancer stem cells and immune-competent models	Lipid ROS, rescue and *in vivo* ferroptosis/immunotherapy analyses	Cross-cancer paradigm requiring breast cancer validation	III

Several widely discussed pathways remain less secure in BCSCs. GPX4, FSP1, SCD1, ACSL3, ACSL4 and ferritinophagy all regulate ferroptosis in breast cancer or other tumors, but their functional contribution to serially transplantable BCSCs is unevenly tested. They should be presented as candidate defenses unless the study demonstrates both ferroptotic rescue and a BCSC endpoint. This distinction matters because an intervention may kill bulk TNBC cells through ferroptosis without preferentially eliminating the residual tumor-initiating compartment.

Several additional regulators are plausible in stem-like breast cancer but remain incompletely validated in that compartment. Lipid-droplet storage and lipophagy can alter the supply of peroxidizable fatty acids (97), whereas SCD1-dependent monounsaturated-fatty-acid synthesis is protective in ovarian cancer (98). Cell-density sensing through Hippo pathway effectors can alter ferroptosis sensitivity, as shown for TAZ and NF2–YAP signaling in non-breast models (99, 105). p53 can also engage SAT1-dependent polyamine metabolism, and NRF2 activation can suppress ferroptosis through a broader antioxidant program (100, 106). These mechanisms should be treated as candidate modifiers of the BCSC ferroptosis threshold, not as established components of a single breast cancer pathway.

### 5.4 Ferroptosis and metastatic plasticity

Metastasis imposes changing ferroptotic pressures. Cells leaving the primary tumor encounter detachment, circulatory oxidative stress, and lipid environments that differ markedly among lung, liver, brain and bone. Mesenchymal states can contain more PUFA-rich membranes and may be relatively sensitive to GPX4 inhibition, yet successful metastasis requires cells to survive this vulnerability. The DKK1–SLC7A11 study provides a concrete solution: BCSC-regulated differentiation raises antioxidant protection during metastatic outgrowth (8). Ferroptosis resistance is therefore coupled to state transition rather than fixed to a single EMT phenotype.

This dynamic view helps reconcile two observations that are often presented as contradictory. EMT and therapy-resistant states can be ferroptosis-sensitive because of their membrane composition, while metastatic lesions are frequently ferroptosis-resistant. Selection during dissemination can enrich cells that retain mesenchymal competence but activate cystine uptake, NRF2, GPX4, FSP1 or MUFA remodeling. The critical trait is the ability to cross the oxidative bottleneck and then re-enter proliferation. CPT1A may contribute by providing energy and redirecting fatty-acid substrates, but direct proof during each metastatic phase is still missing.

Metastatic transit provides a natural selection experiment for ferroptosis. In melanoma, lymph protects disseminating cells from ferroptosis, whereas blood exposure imposes a greater oxidative burden (54). Mesenchymal and drug-tolerant cancer states are often hypersensitive to GPX4 inhibition (55, 56), and differentiation state can predict ferroptosis vulnerability (57). Metabolic heterogeneity also determines which melanoma cells form distant metastases (84). These studies are not breast cancer evidence, but they clarify the question that breast models must answer: does CPT1A protect a metastasis-initiating cell during circulation, during early colonization, or only after local lipids become available? A single endpoint measured in a large established metastasis cannot resolve those stages.

### 5.5 Ferroptosis in dormant and residual breast cancer cells

Dormant disseminated cells survive for long intervals with limited proliferation and fluctuating access to nutrients. Their low biosynthetic rate may reduce peroxide production, but prolonged survival requires efficient membrane maintenance and antioxidant defense. Lipid droplets can sequester fatty acids away from membranes and supply substrates during reactivation; ferritin can buffer iron until lysosomal or autophagic mobilization changes the labile pool. These features create a state that may be resistant at baseline yet vulnerable during exit from dormancy.

Evidence directly connecting CPT1A, ferroptosis and dormancy in breast cancer remains sparse. The greatest vulnerability may arise during reactivation rather than quiescence, when mitochondrial activity, membrane synthesis and iron demand increase together. ER-positive residual disease can acquire oxidative and FAO-dependent features (16, 17, 31), but whether CPT1A inhibition exposes a ferroptotic liability during reactivation remains unknown. This potential therapeutic window should be tested in time-resolved residual-disease and metastatic-relapse models rather than short-term viability assays.

Dormancy adds another temporal constraint. Breast cancer cells can be held quiescent by perivascular thrombospondin-1, escape latency through altered WNT regulation, or acquire dissemination competence in hypoxic primary-tumor regions (76–78). HER2-driven mammary tumors may disseminate at an unexpectedly early stage (79), and single metastatic cells can display a stem-like program before overt outgrowth (71). None of these studies establishes a universal CPT1A-high dormant state. Their implication is narrower but important: the substrate and redox requirements of residual cells are likely to change between entry into dormancy, long-term maintenance and reactivation. CPT1A should be measured longitudinally across those phases rather than assigned a single role in “dormant disease.”

## 6 CPT1A as the metabolic interface between stemness and ferroptosis

### 6.1 The central metabolic partitioning model

Long-chain fatty acids entering a breast cancer cell are distributed among at least three competing fates: mitochondrial oxidation, neutral-lipid storage, and membrane phospholipid synthesis. CPT1A controls access to the first route; DGAT enzymes and lipid droplets buffer the second; ACSL4 and LPCAT3 help create ferroptosis-sensitive phospholipids in the third. The routes are not isolated. Lipid droplets can release substrates for FAO, and inhibition of mitochondrial entry can increase or decrease membrane PUFA content depending on esterification, desaturation and storage capacity.

The central hypothesis of this review is therefore a partitioning hypothesis. CPT1A does not suppress ferroptosis simply because FAO is “antioxidant.” Rather, it may alter the balance between oxidizable membrane substrate, energetic reserve, and the capacity to remove peroxides. This model predicts that CPT1A inhibition will be most ferroptotic when fatty acids are redirected toward ACSL4/LPCAT3-dependent phospholipids and when GPX4/FSP1 defense is limited. If excess fatty acids are safely stored in lipid droplets or converted to MUFAs, the same intervention may not induce ferroptosis.

The partitioning model is a mass-balance hypothesis, not a claim that every molecule excluded from mitochondria enters a ferroptotic phospholipid. Acyl-CoA synthetase specificity, subcellular acyl-CoA pools, lipid-droplet access and phospholipid-remodeling enzymes determine the fate of individual fatty-acid species. Ether lipids and phospholipids carrying two polyunsaturated tails are particularly relevant because they can substantially increase ferroptosis susceptibility (48, 90). Conversely, storage and SCD1-dependent monounsaturated-fatty-acid production can lower membrane peroxidizability (97, 98). The model therefore predicts a change in molecular lipid species, not merely an increase in total lipid after CPT1A inhibition. Stable-isotope tracing into triglycerides and defined phospholipid classes is required to test it.

### 6.2 Energy production and maintenance of stemness

CPT1A-dependent FAO can preserve ATP during radiation, endocrine therapy, detachment and nutrient limitation (10–12, 16, 17). The energetic contribution supports repair, ion gradients, proteostasis and re-entry into the cell cycle. In BCSCs, these functions help maintain self-renewal and tumor initiation, but they are not specific to stemness. The decisive question is whether CPT1A supplies a reserve that competing pathways cannot replace in a particular residual state. Direct flux and rescue experiments should define that dependency before CPT1A is labeled a stem-cell-selective target.

### 6.3 Redox buffering and ferroptosis suppression

The redox arm of the model is indirect and context dependent. AMPK–ACC signaling can preserve NADPH by reducing its consumption in lipogenesis and by permitting oxidative metabolism (29). This mechanism provides cross-context support for the proposed redox consequences of FAO but has not been shown to operate through CPT1A in breast cancer stem cells. FAO may also supply TCA-cycle intermediates to malic enzyme, isocitrate dehydrogenase and one-carbon pathways, although the contribution of these reactions must be measured rather than inferred from FAO alone. In breast cancer, OVOL2-deficient TNBC provides more direct evidence that increased CPT1-dependent FAO can raise NADPH and GSH/GSSG ratios while supporting stemness (28). Because both CPT1A and CPT1B are induced in this model, however, the redox phenotype cannot yet be assigned exclusively to CPT1A.

The same mitochondrial flux can increase ROS. Whether CPT1A raises or lowers ferroptotic pressure depends on electron-transport efficiency, oxygen, iron, and the strength of GPX4-independent defenses. This duality explains why CPT1A should not be placed unconditionally upstream of GPX4 in a linear pathway. It is better represented as a modifier of the redox environment in which GPX4, FSP1 and DHODH operate.

Redox effects of CPT1A are expected to be biphasic. Greater FAO may preserve ATP and provide carbon for auxiliary NADPH-producing reactions, thereby supporting GPX4-dependent peroxide removal (46). The same increase in mitochondrial substrate oxidation can raise electron pressure and ROS, particularly if respiratory capacity, oxygen or iron handling is limiting (50). Moreover, antioxidant remodeling can occur independently through vitamin K, CoQ and NRF2-regulated systems (52, 101). This makes baseline respiration an insufficient predictor of ferroptosis. The informative variable is whether CPT1A perturbation changes the ratio between phospholipid peroxide generation and the combined detoxifying capacity of the cell.

### 6.4 Membrane lipid remodeling

Substrate allocation provides a plausible route from CPT1A to ferroptosis, but the individual steps are supported at different levels. ACSL4 enriches membranes with long-chain ω6 PUFAs and predicts ferroptosis sensitivity in basal-like breast cancer cells (35), whereas ACSL3-dependent incorporation of MUFAs produces a more resistant membrane state (41). These observations establish the importance of phospholipid composition but do not show that CPT1A directly controls either remodeling pathway. We propose that changes in mitochondrial fatty-acid entry may alter the acyl-CoA pools available for storage or phospholipid synthesis. The relevant prediction is not that total cellular PUFA necessarily rises after CPT1A loss, but that the fraction incorporated into defined, oxidation-prone phospholipid species changes. This link remains hypothesis-generating until demonstrated by isotope tracing and lipidomic analysis in breast cancer models.

This issue cannot be resolved by Oil Red O staining or total triglyceride measurement. Required readouts include isotope tracing into acylcarnitines, triglycerides and phospholipid species; quantification of oxidized arachidonoyl/adrenoyl phosphatidylethanolamines; and assessment of lipid-droplet turnover. A rise in lipid droplets after CPT1A inhibition may be protective, harmful or neutral depending on whether PUFA sequestration or lipolytic release dominates.

Membrane architecture is likely to determine whether a change in fatty-acid flux becomes lethal. ACSL4 and LPCAT3 enrich arachidonoyl- and adrenoyl-phosphatidylethanolamines (35, 36), but ferroptosis is not distributed uniformly across all membranes. Mapping studies have identified specific subcellular sites of lipid peroxidation, and phospholipids with two polyunsaturated acyl chains are exceptionally potent substrates (89, 90). Ether-lipid synthesis adds another context-dependent pool (48). In contrast, SCD1 and exogenous monounsaturated fatty acids favor resistant membrane states (41, 98). Lipid droplets can initially sequester fatty acids yet later release them through lipophagy (97). CPT1A will influence ferroptosis only insofar as it changes access to these molecular pools.

### 6.5 CPT1A–c-Myc–NRF2/GPX4 signaling

In lung cancer, CPT1A forms a positive feedback relationship with c-Myc, enhances NRF2/GPX4 signaling, suppresses ACSL4 and supports ferroptosis-resistant cancer stem cells (44). Tumor-associated macrophage-derived L-carnitine further reinforces the pathway, and CPT1A targeting improves immune-checkpoint blockade in mice. This is a compelling mechanistic paradigm because it integrates substrate transport, transcriptional antioxidant defense, membrane remodeling and the immune microenvironment.

It is not yet an established breast cancer pathway. MYC-high TNBC is FAO dependent (21), and NRF2 protects BCSCs from ferroptosis (9), but the complete CPT1A–c-Myc–NRF2/GPX4 circuit has not been demonstrated in the same breast cancer model. The appropriate use of the lung study is to define experiments: test CPT1A–MYC physical and transcriptional feedback, determine whether CPT1A loss changes ACSL4-containing phospholipids, and assess whether NRF2 or GPX4 rescue restores both ferroptosis resistance and tumor initiation.

### 6.6 PPARα–CPT1A-mediated ferroptosis resistance in TNBC

The most direct breast cancer link currently comes from RACGAP1-regulated TNBC (13). RACGAP1 inhibition disrupted PPARα–CPT1A-dependent fatty-acid metabolism, increased lipid peroxidation and ferroptosis, and suppressed tumor growth; restoration of CPT1A partly reversed these effects. The study supports a causal role for CPT1A in ferroptosis resistance in TNBC. It does not establish that the affected cells are BCSCs, and the downstream phospholipid changes remain incompletely resolved. Nevertheless, it provides the breast cancer anchor for the broader gatekeeper hypothesis.

### 6.7 Mitochondrial homeostasis and organelle crosstalk

CPT1A acts within an organelle network. Mitochondria oxidize fatty acids and contain the DHODH–CoQH2 defense; lipid droplets buffer substrates and interact physically with mitochondria; lysosomes mobilize iron and degrade ferritin or lipid droplets. TRPML1 inhibition demonstrates that lysosomal iron handling can determine BCSC ferroptosis (42). Ferritinophagy can raise Fe2+, while lipophagy can release fatty acids that enter either FAO or membrane remodeling. A CPT1A-centered model that excludes lysosomes and lipid droplets is therefore incomplete.

The direction of organelle crosstalk is likely state dependent. During nutrient deprivation, droplet-derived fatty acids may sustain FAO and survival. During ferroptosis induction, the same lipolysis may supply oxidizable PUFAs. Mitochondrial damage can lower ATP and antioxidant capacity, but it may also reduce electron-derived ROS. The net phenotype should be established experimentally rather than assigned from organelle morphology.

Organelle crosstalk prevents a simple mitochondrial-versus-membrane interpretation. Mitochondria determine part of the redox and CoQ landscape (50), lysosomes supply redox-active iron, and autophagic degradation of ferritin can increase the labile iron pool (103, 104). Lipid droplets buffer excess fatty acids but can become a source of peroxidizable substrate during lipophagy (97). These routes may move in opposite directions after CPT1A inhibition: neutral-lipid storage can be protective early, whereas subsequent lipolysis or ferritinophagy can increase ferroptotic pressure. Time-resolved measurements of organelle lipid transfer and iron release are therefore more informative than a single terminal lipid-droplet image.

### 6.8 Integrated CPT1A–ferroptosis–stemness model

The integrated model has four layers. Extracellular fatty acids, adipocytes, lipid-droplet lipolysis, carnitine availability, hypoxia and treatment pressure provide inputs. AMPK–ACC, PPARα/PGC-1α, STAT3, MYC and NRF2 shape CPT1A activity and antioxidant capacity. Fatty acids are then allocated among FAO, storage, MUFA-rich membranes and PUFA-phospholipids. The resulting ATP, redox balance, phospholipid composition and iron-dependent peroxide burden determine whether a cell undergoes ferroptosis or maintains stemness, dormancy and treatment persistence.

The components of this model have different levels of support. CPT1A-dependent stemness and PPARα–CPT1A-mediated ferroptosis resistance have direct support in breast cancer (10–13), whereas treatment-persistent states remain less fully resolved (16, 17, 31). The CPT1A–c-Myc–NRF2/GPX4 circuit derives from lung cancer (44), and CPT1A-dependent redistribution of fatty acids toward oxidation-prone phospholipids remains hypothetical. These distinctions are reflected in the integrated model (**Figure 3**).

**Figure 3:**
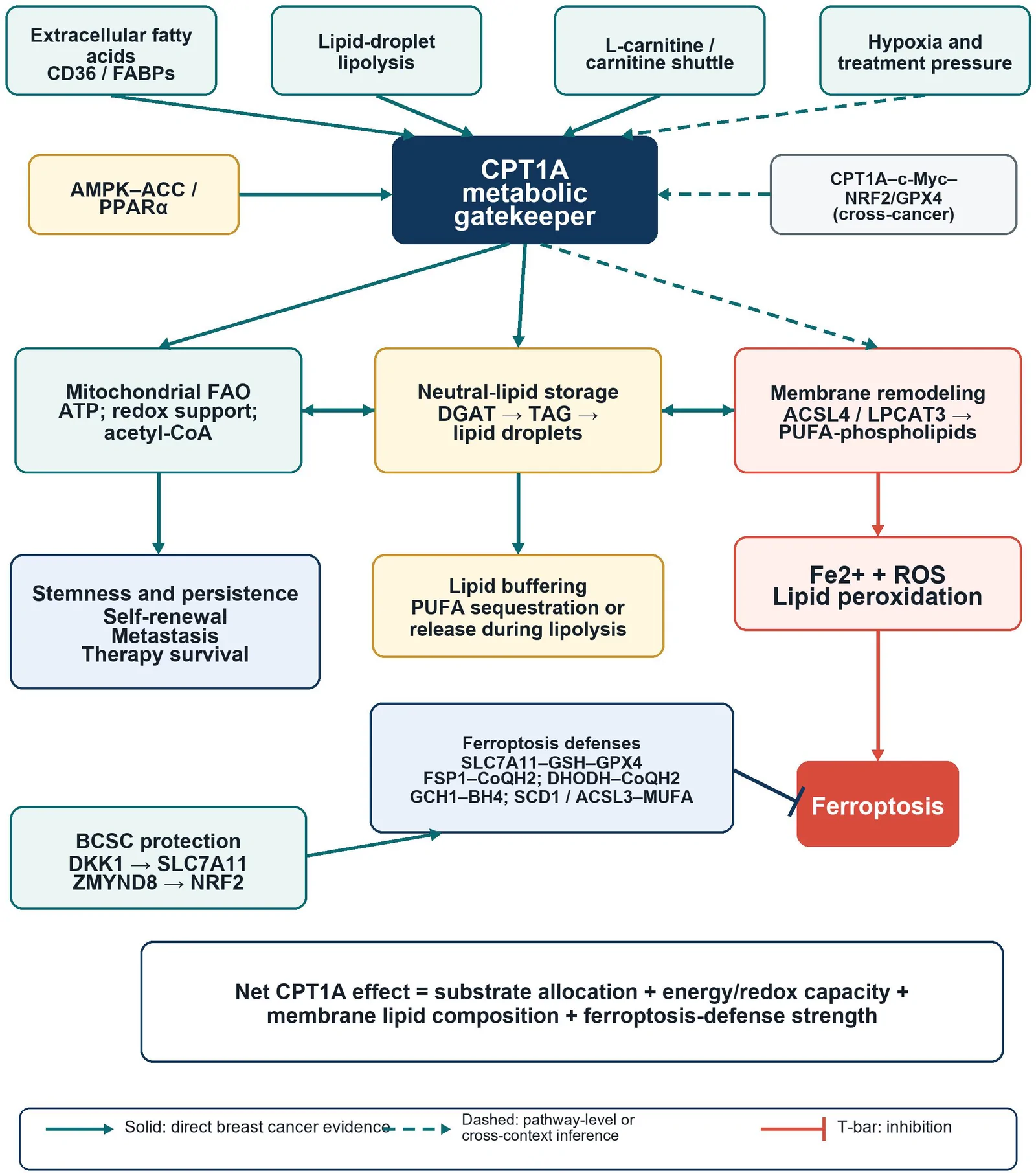
Integrated model of CPT1A as a metabolic gatekeeper linking fatty-acid oxidation, ferroptosis resistance and breast cancer stemness. CPT1A influences competition among mitochondrial oxidation, neutral-lipid storage and PUFA-phospholipid synthesis. The net ferroptosis phenotype depends on substrate allocation, energy/redox capacity, membrane composition and GPX4-dependent and GPX4-independent defenses. The CPT1A–c-Myc–NRF2/GPX4 module is a cross-cancer paradigm requiring validation in breast cancer.

## 7 Context-dependent heterogeneity across breast cancer subtypes and disease states

### 7.1 Triple-negative breast cancer

TNBC contains the strongest convergence of FAO, EMT, stemness and ferroptosis evidence. MYC-high tumors can be FAO dependent (21); OVOL2 loss activates STAT3–CPT1A/CPT1B and tumor initiation (28); FAO supports mesenchymal plasticity (20); and PPARα–CPT1A contributes to ferroptosis resistance (13). Basal-like cells often express ACSL4 and can be ferroptosis sensitive (35), yet BCSC-enriched and metastatic states activate NRF2, SLC7A11 and other defenses (8, 9). These studies establish individual components rather than a unified CPT1A–FAO–ferroptosis pathway. Stratification according to oxidizable phospholipids and active ferroptosis defenses therefore remains a testable framework rather than a validated classification.

TNBC also illustrates why pathway abundance and drug response cannot be conflated. A BET/proteasome synthetic-lethal screen exposed an intrinsic ferroptosis susceptibility in multiple TNBC models through increased labile iron, reduced glutathione and loss of GPX4-associated defenses (58). Conversely, mesenchymal and persister states can remain viable precisely because they become dependent on GPX4 (55, 56). Together with the PPARα–CPT1A study (13), these findings raise the possibility of a peroxidation-prone state and a defense-high state. Whether the latter requires combined CPT1A and GPX4 inhibition has not been tested directly in TNBC and remains a therapeutic hypothesis.

### 7.2 Estrogen receptor-positive breast cancer

ER-positive disease provides strong evidence for CPT1A dependence and therapy-induced FAO. CPT1A is amplified or overexpressed in a subset of aggressive luminal tumors and supports mammosphere formation (11). c-Jun, FDXR and AMPK–Src programs increase CPT1A/FAO during endocrine resistance (16, 17, 31). Direct links to ferroptosis are less developed. A key translational question is whether CPT1A-high endocrine-resistant tumors also acquire GPX4 or SLC7A11 dependence, creating a combination vulnerability. The long natural history of ER-positive disease makes residual and dormant models particularly important.

### 7.3 HER2-positive breast cancer

HER2-positive tumors are metabolically active and undergo substantial lipid reprogramming after targeted therapy. PPARG-driven fatty-acid metabolism has been associated with resistance to trastuzumab, lapatinib and pyrotinib (32), and TRPML1 inhibition can eliminate stem-like cells in HCC1954 models through ferroptosis (42). Direct evidence joining CPT1A, ferroptosis and HER2-positive BCSCs remains limited. This subtype should therefore be presented as a priority for validation, not as an established CPT1A-dependent population.

### 7.4 Primary tumors, circulating cells, and metastatic lesions

The substrate environment changes across disease compartments. Primary breast tumors receive fatty acids from adjacent adipocytes; circulating cells face detachment and oxidative stress; lung and liver metastases encounter distinct lipid and oxygen landscapes; brain metastases must adapt to the blood–brain barrier and neural lipids; bone lesions occupy a hypoxic, iron- and stromal-cell-rich niche. A CPT1A-high state in one compartment may not persist in another. Paired primary-metastatic sampling and organ-specific models are needed to define when the gatekeeper operates.

Metastatic-site biology should be treated as more than an anatomic variable. Early breast cancer metastases can be enriched for a stem-like transcriptional program (71), and perivascular, hypoxic and immune niches regulate persistence (76–79). CD36-dependent lipid use supports metastasis-initiating cells in squamous carcinoma (72), while lymph lipids and cell-intrinsic metabolic heterogeneity alter ferroptosis survival and metastatic efficiency in melanoma (54, 84). Adipocyte-derived fatty acids promote growth in breast and ovarian models (81–83). These cross-tumor findings define plausible pressures but not a breast-specific CPT1A mechanism. Direct comparison of paired primary tumors, circulating cells and organ-matched metastases is needed.

### 7.5 Obesity and systemic metabolic status

Obesity increases circulating fatty acids, adipokines and inflammatory signals and expands the breast adipose compartment. Adipocyte-derived leptin can activate STAT3–CPT1B–FAO in BCSCs (7), and related signals may alter CPT1A in other contexts. Menopause, insulin resistance, dietary fatty-acid composition and systemic carnitine availability are potential modifiers. None should be assumed to predict ferroptosis resistance without tumor lipidomics and flux measurements. **Table 4** summarizes subtype- and state-specific evidence and the principal unresolved questions (**Table 4**).

**Table 4:** Context-specific CPT1A and ferroptosis dependencies across breast cancer states.

Clinical context	CPT1A/FAO phenotype	Ferroptosis/stemness phenotype	Potential intervention	Evidence status
Triple-negative breast cancer	MYC-, STAT3- or PPARα-associated FAO; CPT1A/CPT1B context dependent	ACSL4-associated vulnerability opposed by NRF2/SLC7A11 defenses; EMT plasticity	Biomarker-guided CPT1A/FAO inhibition plus ferroptosis induction	Strongest convergence; still state dependent
ER-positive/endocrine-resistant disease	CPT1A amplification or induction through c-Jun, FDXR and AMPK–FAO–Src	Long-lived oxidative persistence; direct ferroptosis linkage limited	Metabolic priming with endocrine/Src therapy; test GPX4/system xc− combinations	Strong CPT1A/FAO evidence; BCSC-ferroptosis gap
HER2-positive disease	PPARG/fatty-acid metabolism after anti-HER2 therapy; CPT1A role unresolved	TRPML1-sensitive stem-like cells; direct CPT1A evidence sparse	Validate CPT1A dependence; lysosomal ferroptosis strategies	Priority evidence gap
Radiation-selected residual disease	CPT1A/CPT2 and FAO increased	Stem-like, clonogenic and radioresistant phenotype	Sequential CPT1A/FAO inhibition after radiotherapy	Direct breast cancer evidence
Obesity/adipocyte-rich niches	Leptin–STAT3–CPT1B and increased lipid supply	BCSC self-renewal and chemoresistance	Target FAO plus adipocyte/leptin signaling; define CPT1 isoform	Direct CPT1B evidence; CPT1A extrapolation limited

## 8 Therapeutic targeting of the CPT1A–FAO–ferroptosis axis

### 8.1 Direct CPT1A inhibition

Etomoxir is widely used to inhibit CPT1, but at common experimental concentrations it perturbs CoA homeostasis and mitochondrial respiration independently of CPT1A (19, 23). A manuscript that relies on etomoxir alone cannot establish CPT1A dependence. Perhexiline and teglicar have different pharmacological profiles but are not tumor-selective CPT1A drugs. Strong preclinical studies should combine low-dose pharmacology with CPT1A knockdown or knockout, wild-type rescue, and ideally a catalytically inactive mutant.

The therapeutic challenge is selectivity. CPT1A supports hepatic, immune and other normal-cell metabolism, and systemic inhibition may alter antitumor immunity as well as tumor-cell fitness. RNA delivery, antibody-targeted nanoparticles and tumor-selective degradation strategies may provide a wider window than chronic systemic enzyme inhibition. Intermittent scheduling is another rational approach because residual tumor cells may depend on FAO only during defined treatment transitions.

### 8.2 Ferroptosis-inducing approaches

Ferroptosis can be induced by limiting cystine uptake, depleting glutathione, inhibiting GPX4, increasing the labile iron pool, or enriching oxidizable phospholipids. Erastin-related compounds and sulfasalazine target system xc−; RSL3, ML162 and ML210 inhibit GPX4 in experimental systems; lysosomal interventions can mobilize iron; and SCD1/FSP1 inhibition can remove GPX4-independent protection. None of the canonical GPX4 inhibitors is currently a routine systemic anticancer drug, largely because of pharmacokinetic and toxicity limitations.

BCSC-directed evidence favors mechanisms that create a state-specific liability. TRPML1 inhibition disrupts lysosomal iron handling and sensitizes BCSCs to doxorubicin (42). Interfering with hypoxic cysteine production exposes a BCSC ferroptosis vulnerability (43). DKK1 blockade plus ferroptosis induction suppresses metastatic outgrowth (8). These approaches may offer greater selectivity than indiscriminate iron delivery.

### 8.3 Rational combination strategies

CPT1A inhibition plus GPX4 inhibition is mechanistically attractive when CPT1A loss redirects PUFAs toward membranes and reduces redox reserve. The combination should not be assumed to be synergistic; it must be tested with lipidomics and rescue. CPT1A inhibition plus system xc− blockade may be particularly effective in cystine-dependent residual cells. In TNBC, PPARα–CPT1A disruption already increases ferroptosis (13), providing a direct basis for combination studies.

Radiotherapy is a second logical partner. Radiation selects a CPT1A/CPT2-high, FAO-dependent state (12) and simultaneously produces oxidative stress. Sequential CPT1A inhibition after radiation may prevent recovery while avoiding interference with initial oxygen-dependent damage. In ER-positive disease, metabolic priming with CPT1 inhibition can enhance endocrine and Src-targeted therapy (31). Chemotherapy combinations should account for isoform context because adipocyte-supported BCSCs may be CPT1B dominant (7).

Immunotherapy requires special caution. The lung cancer study suggests that CPT1A targeting can promote tumor ferroptosis and improve checkpoint blockade (44), but systemic FAO inhibition may also affect T cells and myeloid cells. Breast cancer studies must separate tumor-cell and host-cell CPT1A genetically before advancing the combination. The same principle applies to macrophage-derived carnitine and adipocyte-derived lipids.

The immunological consequences of this combination are genuinely bidirectional. Activated CD8+ T cells can promote tumor-cell ferroptosis through interferon-γ-dependent repression of system xc− (59). Yet oxidized-lipid uptake through CD36 drives ferroptosis and dysfunction in intratumoral CD8+ T cells (60, 61). Myeloid metabolism adds further complexity: FATP2-mediated arachidonic-acid uptake reprograms suppressive neutrophils, and FAO supports suppressive functions of myeloid-derived suppressor cells (93, 102). Memory CD8+ T cells, by contrast, use cell-intrinsic lipolysis to support their metabolic program (94). Systemic CPT1A or lipid-oxidation inhibition could therefore improve one compartment while compromising another. Tumor-cell-restricted perturbation and immune-competent models are mandatory.

Ferroptotic cell death is not uniformly immunogenic. Vaccination with cells collected at an early ferroptotic stage generated antitumor immunity in one model (95), whereas ferroptotic cells in another system impaired dendritic-cell maturation, uptake and antigen cross-presentation (96). Differences in inducer, timing and the stage at which dying cells were harvested probably contribute to the discrepancy. For CPT1A–ferroptosis combinations, this argues against using tumor shrinkage alone as evidence of immune synergy. Studies should define the kinetics of lipid peroxidation and membrane rupture, quantify antigen-presenting-cell function and compare simultaneous with sequential checkpoint blockade.

### 8.4 Targeted delivery to breast cancer stem cells

Targeted delivery can reduce normal-tissue exposure and concentrate combinations in residual niches. Hyaluronic-acid/CD44 systems, EpCAM-directed carriers, lipid nanoparticles and microenvironment-responsive polymers are plausible platforms for CPT1A siRNA, ferroptosis inducers or both. Marker heterogeneity is a limitation: CD44high/CD24low and ALDHhigh states interconvert, so a single-marker carrier may spare the alternate state. Dual targeting or delivery driven by metabolic features, such as high lipid uptake, may be more robust.

### 8.5 Treatment sequence and timing

Sequence is part of the mechanism. CPT1A inhibition before therapy may prevent metabolic adaptation but increase toxicity in normal tissues. Administration after radiation or chemotherapy may preferentially attack the selected residual population. Ferroptosis induction after CPT1A inhibition may allow time for membrane lipid remodeling; simultaneous dosing may not. Preclinical designs should measure the temporal sequence of FAO suppression, phospholipid remodeling, lipid ROS and BCSC depletion. **Table 5** summarizes therapeutic strategies, evidence and major limitations (**Table 5**).

**Table 5:** Therapeutic strategies targeting the CPT1A–FAO–ferroptosis axis.

Strategy	Mechanistic rationale	Breast cancer evidence	BCSC evidence	Major limitation/biomarker
Direct CPT1A inhibition	Restrict long-chain fatty-acid entry and metabolic reserve	Genetic and pharmacological studies in luminal, TNBC, radiation and endocrine resistance	Direct in selected models (10–12, 28)	Isoform specificity, etomoxir/perhexiline off-target effects; require FAO flux
CPT1A + GPX4 inhibition	Reduce metabolic buffering while blocking peroxide detoxification	Rationale supported by TNBC CPT1A–ferroptosis evidence (13)	Not yet established in functional BCSCs	Normal-tissue toxicity; select CPT1A-high/GPX4-high tumors
CPT1A + system xc− inhibition	Limit redox adaptation and cysteine/GSH supply	Mechanistically plausible; SLC7A11 important in metastasis (8)	Direct SLC7A11 BCSC-related evidence	Cystine availability and compensatory transsulfuration
CPT1A/FAO inhibition + radiotherapy	Block radiation-induced FAO adaptation	Direct CPT1A/CPT2 evidence (12)	Radiation-derived BCSCs studied	Sequence and host-tissue radiosensitization
CPT1A/FAO inhibition + endocrine/Src therapy	Reverse c-Jun/FDXR/AMPK–FAO–Src resistance	Multiple ER-positive models (16, 17, 31)	Stemness-specific evidence incomplete	Select endocrine-resistant FAO-high tumors
TRPML1 inhibition + chemotherapy	Disrupt lysosomal iron control and induce ferroptosis	Breast cancer models with doxorubicin (42)	Direct BCSC depletion and sphere/tumor suppression	Lysosomal toxicity and delivery
CPT1A targeting + immunotherapy	Promote tumor ferroptosis and relieve immune suppression	Strong lung cancer paradigm (44); breast validation lacking	Cross-cancer CSC evidence	Separate tumor-cell and immune-cell CPT1A

## 9 Biomarkers and strategies for patient selection

### 9.1 Tumor-cell biomarkers

CPT1A expression is a starting point, not a sufficient biomarker. A useful panel should include CPT1A/CPT2, PPARα or PGC-1α, CD36/FABPs, and the ferroptosis-defense proteins GPX4, SLC7A11, FSP1 and NRF2. ACSL4 and LPCAT3 report potential PUFA-phospholipid synthesis, while SCD1 and ACSL3 indicate MUFA protection. ALDH1A1 and CD44/CD24 can enrich stem-like states but should not be interpreted independently of function.

### 9.2 Functional metabolic biomarkers

Functional measurements are more informative than single transcripts. Candidate assays include 13C-palmitate oxidation, acylcarnitine profiling, palmitate-supported respiration, PUFA/MUFA phospholipid ratios, oxidized phospholipid species, GSH/GSSG, lipid ROS and the labile iron pool. Patient-derived organoids may permit ex vivo testing of CPT1A and ferroptosis combinations. A clinically practical assay will probably combine tissue protein markers with plasma acylcarnitines or lipid products rather than reproduce full flux analysis.

A useful biomarker must also be mechanistically proximal. Transcript abundance can identify a candidate state, but it cannot establish that fatty acids reach mitochondria or that a membrane contains the phospholipids that actually oxidize. Genome-scale ferroptosis screens, chemical-genetic analysis and subcellular lipid-peroxidation mapping have identified dependencies that would be missed by a CPT1A/GPX4 expression pair alone (85–90). Practical patient selection may therefore require a layered assay: tumor-cell protein localization, targeted phospholipid and acylcarnitine measurements, and a short ex vivo perturbation in an organoid or fresh slice. The signature proposed below should be judged against such functional endpoints rather than against prognosis alone.

### 9.3 Proposed CPT1A–ferroptosis–stemness signature

We propose a research signature with three modules: a CPT1A/FAO module (CPT1A, CPT2, PPARA, PPARGC1A and ACADVL), a ferroptosis-defense module (GPX4, SLC7A11, AIFM2, NFE2L2 and GCH1), and a stemness module (ALDH1A1, CD44, SOX2, NANOG and POU5F1). The signature is intended to identify CPT1A-high tumors that also possess strong peroxide defense and stem-like features. It is not a validated clinical score.

**Figure 4** presents a hypothesis-generating stratification framework (**Figure 4**). CPT1A-high/defense-high tumors may require combined metabolic and ferroptosis targeting; ACSL4-high/defense-low tumors may already be ferroptosis-prone; endocrine-resistant FAO-high tumors may benefit from metabolic priming; and therapy-induced FAO-high residual disease may require timed intervention. Each group must be validated prospectively.

**Figure 4:**
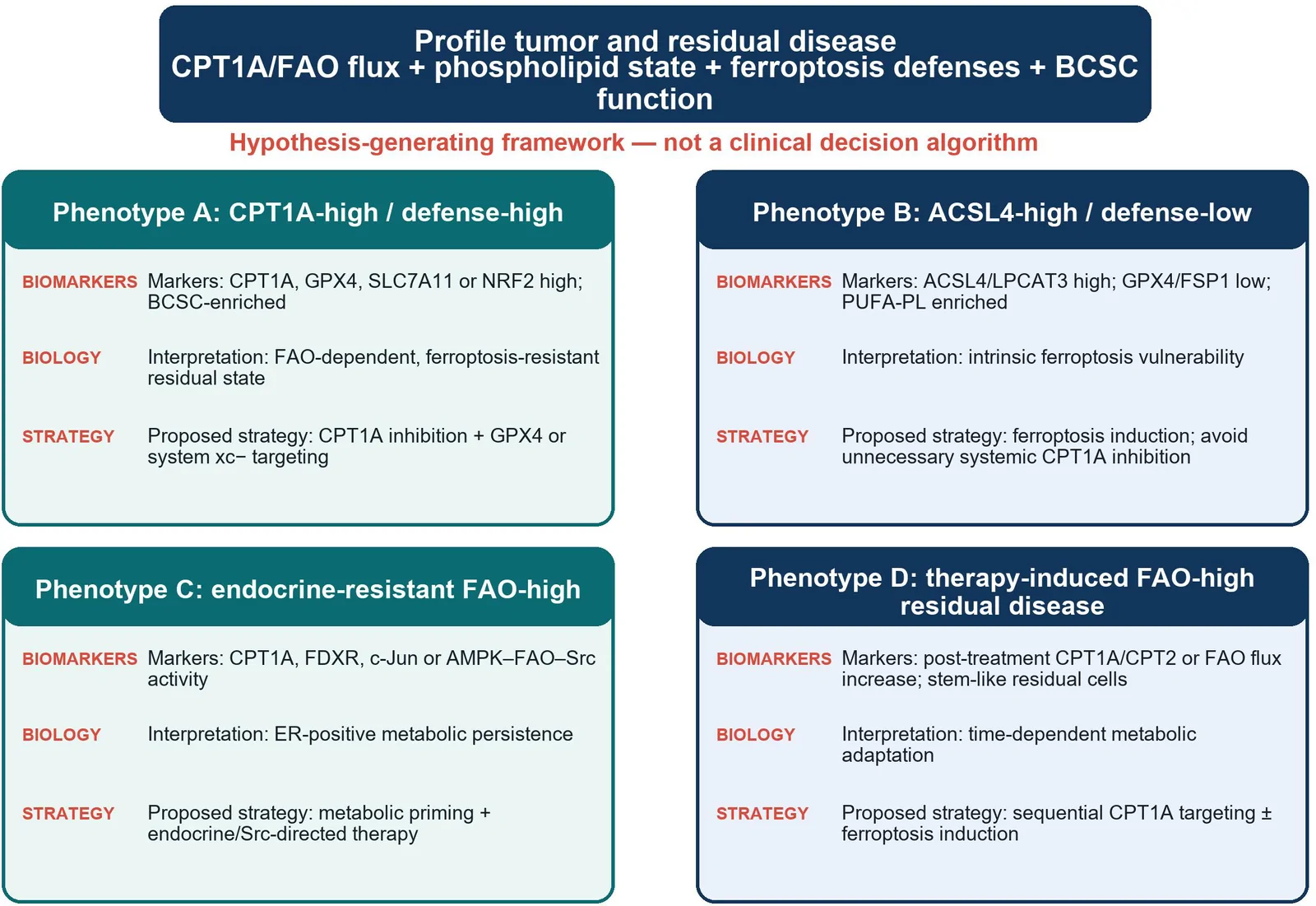
Biomarker-guided therapeutic stratification of the CPT1A–ferroptosis axis in breast cancer. Four hypothesis-generating metabolic phenotypes integrate CPT1A/FAO activity, ferroptosis defenses, phospholipid composition, treatment history and BCSC enrichment. The framework is intended to guide experimental validation and is not a clinical decision algorithm.

### 9.4 Liquid biopsy and metabolic imaging

Circulating tumor cells could reveal whether CPT1A-high stem-like states persist during treatment. Extracellular-vesicle RNA, plasma acylcarnitines and oxidized lipid products are candidate pharmacodynamic markers, although none is tumor specific. Metabolic imaging of fatty-acid use remains investigational. Longitudinal sampling may be more informative than a baseline measurement because the pathway is treatment inducible.

## 10 Controversies, methodological limitations, and unanswered questions

### 10.1 Does CPT1A always suppress ferroptosis?

No. CPT1A can support antioxidant capacity and remove fatty acids from the membrane precursor pool, but it can also increase electron flux and ROS. The outcome depends on substrate type, phospholipid remodeling, oxygen, iron and defense systems. Any universal arrow from CPT1A to ferroptosis suppression is an oversimplification.

### 10.2 Is FAO activity equivalent to CPT1A expression?

No. CPT1A abundance does not establish transport or oxidation, and FAO can involve CPT1B or medium-chain substrates that bypass CPT1. Required measurements include flux, substrate-supported respiration and genetic rescue. Expression-only studies provide biological association, not metabolic dependence.

### 10.3 Off-target limitations of pharmacological inhibitors

High-dose etomoxir disrupts CoA and respiration independently of CPT1A (19, 23). Perhexiline affects additional mitochondrial targets and has a narrow therapeutic index. Pharmacological phenotypes should agree with isoform-specific genetics and be rescued by CPT1A re-expression.

### 10.4 Are breast cancer stem-cell markers sufficiently specific?

CD44high/CD24low, ALDHhigh, mammosphere formation and stemness transcription factors identify overlapping but non-identical states. Mammospheres are influenced by aggregation and proliferation. Claims of self-renewal should incorporate serial passage, limiting-dilution transplantation, lineage tracing or relapse assays.

### 10.5 A minimal checklist for confirming ferroptosis

Changes in GPX4, ACSL4, total ROS or viability are insufficient to establish ferroptosis. At minimum, studies should demonstrate lipid peroxidation and iron dependence, rescue with ferrostatin-1 or liproxstatin-1 and an iron chelator, and exclusion of apoptosis and other major cell-death pathways. Oxidized phospholipid lipidomics provides additional biochemical support.

### 10.6 Tumor-cell versus immune-cell CPT1A

Systemic CPT1A inhibition affects tumor cells, macrophages, T cells, adipocytes and liver. Host effects may reinforce or oppose tumor-cell ferroptosis. Compartment-specific knockout and immune-competent models are essential before interpreting immunotherapy combinations.

The host-cell problem is not a secondary toxicity issue; it can reverse the biological interpretation of treatment. CD8+ T cells may either induce tumor ferroptosis or become ferroptotic themselves depending on cytokine signaling and oxidized-lipid uptake (59–61). Suppressive neutrophils and myeloid-derived suppressor cells use fatty-acid transport and oxidation programs that can be therapeutically favorable to disrupt (93, 102), whereas memory T cells require lipid mobilization for durable function (94). A systemic metabolic inhibitor therefore changes both sides of the tumor–immune interaction. Conditional tumor and host knockouts, bone-marrow chimeras and compartment-resolved pharmacodynamic measurements should precede claims of immune synergy.

### 10.7 Normal-tissue toxicity and therapeutic window

Long-chain FAO is important in liver and other normal tissues; related CPT1 isoforms are critical in heart and muscle. Ferroptosis induction can damage kidney, nervous system and immune cells. Tumor-selective delivery, intermittent treatment and pharmacodynamic monitoring will be required to obtain a therapeutic window.

## 11 Future research roadmap

### 11.1 Establish direct causality

The central experiment is straightforward in concept: manipulate CPT1A in functionally defined BCSCs, quantify FAO and oxidized phospholipids, and determine whether ferroptosis rescue restores self-renewal and tumor initiation. Wild-type and catalytically inactive rescue should distinguish transport-dependent from noncanonical functions. The same design should be repeated in TNBC, ER-positive and HER2-positive models.

### 11.2 Separate canonical and noncanonical CPT1A functions

CRISPR knockout, mitochondrial-localization mutants and catalytic mutants are needed to test whether stemness depends on acylcarnitine formation. Proposed succinyltransferase or protein-interaction functions should be evaluated independently of FAO. Drug and genetic phenotypes must converge.

### 11.3 Perform isotope-resolved metabolic flux analysis

13C-palmitate, 13C-oleate and 13C-linoleate should be traced simultaneously into acylcarnitines, TCA intermediates, triglycerides and membrane phospholipids. Oxidized lipid species should be measured rather than inferred. This will test the partitioning model directly.

A decisive flux experiment should follow several fatty-acid species in parallel. Palmitate reports saturated-fatty-acid oxidation but cannot test whether CPT1A depletion redirects oleate, linoleate or arachidonate toward protective triglycerides, ether lipids or highly peroxidizable phospholipids. The same samples should quantify acylcarnitines, TCA labeling, neutral lipids, one- and two-PUFA phospholipids, and oxidized products (48, 89, 90). Ferritin turnover and lipophagy should be measured over the same time course because iron release and fatty-acid mobilization can lag behind the initial perturbation (97, 103, 104). Only this integrated design can distinguish substrate redistribution from nonspecific oxidative collapse.

### 11.4 Integrate single-cell and spatial technologies

Single-cell RNA and chromatin data can identify CPT1A-high states, but spatial lipidomics and multiplex protein imaging are required to define substrate context. The key niches are CPT1Ahigh/ALDH1A1high/GPX4high regions near adipocytes, macrophages or treatment-damaged tissue. Longitudinal sampling should determine whether these states pre-exist or emerge after therapy.

### 11.5 Use clinically relevant models

Priority models include patient-derived organoids, residual-disease patient-derived xenografts (PDXs), spontaneous metastasis models, immune-competent tumors, obesity-associated models, and organ-specific lung, bone, liver and brain metastases. Short-term monolayer viability is insufficient for a question centered on recurrence.

### 11.6 Develop biomarker-guided clinical strategies

Preclinical studies should define a pharmacodynamic signature of CPT1A inhibition and establish whether CPT1A-high/GPX4-high tumors respond to combination therapy. Plasma acylcarnitines, tissue lipidomics and dynamic BCSC markers should be evaluated together.The immediate priority is an isoform-resolved, time-resolved test of whether CPT1A inhibition redirects defined fatty acids into oxidation-prone phospholipids and eliminates tumor-initiating cells through ferroptosis. **Table 6** lists the principal knowledge gaps and the experiments required to close them (**Table 6**).

**Table 6:** Critical knowledge gaps and recommended experimental solutions.

Knowledge gap	Why current evidence is insufficient	Recommended experiment/model	Required readout	Translational value
Does CPT1A directly control BCSC ferroptosis?	Stemness and ferroptosis are rarely tested in the same model	CPT1A knockout with wild-type/catalytic-mutant rescue in sorted BCSCs and organoids	FAO flux, oxidized phospholipids, ferroptosis rescue, serial tumor initiation	Establishes the central causal claim
Which CPT1 isoform is dominant?	CPT1B or aggregate CPT1 activity is often attributed to CPT1A	Isoform-specific CRISPR and rescue across subtypes	Acylcarnitines, OCR and functional BCSC frequency	Prevents biomarker and drug-selection errors
How are fatty acids repartitioned?	Expression and lipid-droplet staining do not measure fate	Parallel 13C-palmitate/oleate/linoleate tracing	TCA, TAG, phospholipid and oxidized-PE labeling	Tests the gatekeeper model
Pre-existing versus induced dependence	Most resistant models are endpoint comparisons	Longitudinal barcoding and pre/post-treatment single-cell/spatial studies	CPT1A state dynamics and relapse competence	Defines treatment timing
Tumor versus host CPT1A	Systemic inhibition affects immune and metabolic tissues	Compartment-specific knockouts in immune-competent models	Tumor ferroptosis, immune function and toxicity	Determines combination safety
Organ-specific metastatic dependence	Primary-tumor models do not reproduce distant lipid niches	Spontaneous lung, bone, liver and brain metastasis models	Flux, lipidomics and organ-specific relapse	Guides site-specific therapy
Patient-selection biomarker	CPT1A expression alone is not functional dependence	Prospective organoid/PDX validation of a composite signature	Response, pharmacodynamic acylcarnitines and lipid ROS	Enables biomarker-led trials

## 12 Conclusions

CPT1A-mediated fatty-acid oxidation supports stemness, metastatic adaptation and treatment persistence in defined breast cancer contexts. BCSCs also possess ferroptosis liabilities and activate defenses through SLC7A11, NRF2, lysosomal iron control and alternative cysteine supply. These fields intersect at fatty-acid partitioning: CPT1A can influence whether long-chain fatty acids are oxidized, stored, or incorporated into peroxidation-prone phospholipids.

The evidence does not yet justify calling CPT1A a universal master regulator of BCSC ferroptosis. Direct breast cancer support is strongest for CPT1A-dependent stemness in miR-328-3p, luminal and radioresistant models, and for CPT1A-mediated ferroptosis resistance downstream of PPARα in TNBC. The complete CPT1A–ferroptosis–stemness circuit remains to be demonstrated in a single functionally rigorous model. Establishing that causal chain, resolving subtype and host-cell effects, and developing tumor-selective combinations will determine whether the proposed gatekeeper becomes a clinically actionable vulnerability.
